# At Least Ten Genes Define the Imprinted *Dlk1-Dio3* Cluster on Mouse Chromosome 12qF1

**DOI:** 10.1371/journal.pone.0004352

**Published:** 2009-02-05

**Authors:** John P. Hagan, Brittany L. O'Neill, Colin L. Stewart, Serguei V. Kozlov, Carlo M. Croce

**Affiliations:** 1 Department of Molecular Virology, Immunology and Medical Genetics, Comprehensive Cancer Center, The Ohio State University Medical Center, Columbus, Ohio, United States of America; 2 Cancer and Developmental Biology Laboratory, Center for Cancer Research, National Cancer Institute at Frederick, Frederick, Maryland, United States of America; 3 Center for Advanced Preclinical Research and Mouse Cancer Genetics Program, SAIC-Frederick, Inc., National Cancer Institute at Frederick, Frederick, Maryland, United States of America; Texas A&M University, United States of America

## Abstract

**Background:**

Genomic imprinting is an exception to Mendelian genetics in that imprinted genes are expressed monoallelically, dependent on parental origin. In mammals, imprinted genes are critical in numerous developmental and physiological processes. Aberrant imprinted gene expression is implicated in several diseases including Prader-Willi/Angelman syndromes and cancer.

**Methodology/Principal Findings:**

To identify novel imprinted genes, transcription profiling was performed on two uniparentally derived cell lines, androgenetic and parthenogenetic primary mouse embryonic fibroblasts. A maternally expressed transcript termed Imprinted RNA near *Meg3*/*Gtl2* (*Irm*) was identified and its expression studied by Northern blotting and whole mounts *in situ* hybridization. The imprinted region that contains *Irm* has a parent of origin effect in three mammalian species, including the sheep *callipyge* locus. In mice and humans, both maternal and paternal uniparental disomies (UPD) cause embryonic growth and musculoskeletal abnormalities, indicating that both alleles likely express essential genes. To catalog all imprinted genes in this chromosomal region, twenty-five mouse mRNAs in a 1.96Mb span were investigated for allele specific expression.

**Conclusions/Significance:**

Ten imprinted genes were elucidated. The imprinting of three paternally expressed protein coding genes (*Dlk1*, *Peg11*, and *Dio3*) was confirmed. Seven noncoding RNAs (*Meg3/Gtl2*, *Anti-Peg11*, *Meg8*, *Irm/“Rian”*, AK050713, AK053394, and *Meg9/Mirg*) are characterized by exclusive maternal expression. Intriguingly, the majority of these noncoding RNA genes contain microRNAs and/or snoRNAs within their introns, as do their human orthologs. Of the 52 identified microRNAs that map to this region, six are predicted to regulate negatively *Dlk1*, suggesting an additional mechanism for interactions between allelic gene products. Since several previous studies relied heavily on *in silico* analysis and RT-PCR, our findings from Northerns and cDNA cloning clarify the genomic organization of this region. Our results expand the number of maternally expressed noncoding RNAs whose loss may be responsible for the phenotypes associated with mouse pUPD12 and human pUPD14 syndromes.

## Introduction

Unlike other vertebrates, mammalian embryos have an absolute dependency on both parental genomes for successful development. Mouse embryos, in which the entire genome is of maternal (parthenogenetic) or paternal (androgenetic) origin, do not develop past embryonic day 10 [1], [2]. The basis for this developmental failure is that some genes are subject to genomic imprinting and are expressed from only one parental allele. Consequently, loss of the expressed allele will render the embryo null for the imprinted gene's function, or conversely, duplication of the expressed allele may lead to overabundance of the imprinted product leading to detrimental effects. The primary goal of our research was to identify and characterize novel imprinted genes that may contribute to mammalian phenotypes displaying parent of origin effects.

In addition to its developmental roles, appropriate imprinting is important to human health into adulthood, as misexpression of imprinted genes has been implicated in a range of diseases. Imprinted genes that serve roles in growth regulation are often aberrantly expressed in cancers [3], [4]. For example, loss of imprinting that leads to enhanced expression of the insulin-like growth factor 2 (*Igf2*) is frequently observed during cancer progression [5], [6]. Imprinting has also been implicated in a variety of neurological disorders, of which the best characterized are Angelman and Prader-Willi syndromes [7]. More recently, it was shown that the major autism susceptibility locus (AUTS1) on human 7q may have a bias towards paternal inheritance, suggesting the involvement of imprinting [8], [9]. In rodents, female mice deficient in Paternally expressed gene 1/Mesoderm specific transcript (*Peg1/MEST*) [10] or Paternally expressed gene 3 (*Peg3*) [11] have aberrant maternal nurturing behavior with low incidences of pup retrieval, nest building, and milk ejection. These findings clearly illustrate the necessity to gain a better understanding of imprinting in mammalian biology. To achieve this goal, an extensive screen for novel imprinted genes has been undertaken.

In the mouse, most imprinted genes are found in clusters that are distributed among eleven regions on eight chromosomes [12]. These areas were defined by using Robertsonian translocations to generate embryos that are uniparental for specific chromosomal regions that cause obvious defects in development, post-natal growth, and behavior [13]. Many imprinted genes have been identified within these regions [12], [14]. Although the translocation studies may have been exhaustive in defining regions whose uniparental inheritance resulted in overt phenotypes, not all imprinted mouse genes map to these regions. The imprinted mouse genes, *Zac1* [15], *Ins1* [16], *Grf1* [17], *Peg1/MEST* [18], *Nnat* [19], *Htr2* [20], and *Impact* [21], are known to lie outside these regions on seven different chromosomes forming “microimprinted domains,” revealing that imprinted genes are more widely distributed than previously anticipated. Given the significance of imprinting, the identification of other imprinted genes is both necessary and informative, as these findings may provide further insights into the roles of imprinting in mammalian development, behavior, and its contribution to human pathological conditions. Moreover, the elucidation about why and how this unusual form of mammalian epigenetic gene regulation evolved requires additional insights from a more complete analysis of all imprinted genes and regions.

The derivation of androgenetic and parthenogenetic mouse embryonic fibroblast (PMEF) lines that stably retain the parent-of-origin pattern of imprinted gene expression has been previously described. These lines were used as source of material for a subtractive screen for maternally imprinted genes that led to the identification of two novel imprinted genes, ε-sarcoglycan (*Sgce*) and *Zac1*, mapping to mouse chromosomes 6 and 10, respectively [15]. Concomitantly, experiments to determine the effects of DNA methylation and histone acetylation on the maintenance of the imprinted status of gene expression employed these cell lines [22]. Here, we utilized these cell lines in conjunction with DNA microarray technology to search for additional novel imprinted transcripts. We report the isolation and characterization of a maternally expressed RNA called *Irm* (Imprinted RNA near *Meg3*) that is located on the distal region of mouse chromosome 12 near the known imprinted genes, Delta-like homolog 1 (*Dlk1*) and Maternally expressed gene 3/Gene trap locus 2 (*Meg3/Gtl2*). This chromosomal segment has been identified in three different mammalian species - humans, mice, and sheep - as having significant parent of origin effects on embryogenesis, muscle growth and possibly post-natal behavior. In both sheep and humans, this region contains six described genes: *Dlk1*, *Dlk1 associated transcript “DAT”*, *Meg3/Gtl2*, *Peg11*, *Peg11 antisense transcript*, and *Meg8* [23]. In mice, there are six imprinted transcripts for which cDNAs have been cloned. *Meg3/Gtl2* and *Dlk1* were the first two imprinted genes found in this gene cluster [24], [25], [26], [27], [28]. Two noncoding maternally expressed RNAs, *Rian* [29] and the *C/D snoRNA* MBII-343 [30], were discovered later that map to this region, although their precise chromosomal location was unknown at the time. *Dio3* displays a bias for paternal expression, although the maternal allele is expressed at roughly one-quarter of the level of its paternal counterpart [31], [32], [33]. Finally, MicroRNA containing gene (*Mirg*) was identified as another maternally expressed noncoding RNA [34]. To date, cDNA clones have not been isolated for any other imprinted genes in this region.

MicroRNAs are an abundant class of endogenously expressed noncoding RNAs that are 19–24 nucleotides in length. Previously, we have shown that microRNAs can act as cancer classifiers [35], [36] and that microRNAs that are differentially regulated are important in regulating the levels of both oncogenes and tumor suppressors in this disease [37], [38]. Recently implicated directly in cancer initiation and/or progression using mouse models (reviewed in [39]), microRNAs regulate gene expression usually through imperfect base-pairing interactions with the 3′ UTR of its target mRNAs leading to mRNA degradation and/or translational inhibition. Large numbers of mRNAs, often numbering in the hundreds, were shown to be most likely downregulated directly by each of twenty-six microRNAs investigated [40], [41]. As would be expected for a potent gene regulator, mouse knockouts of miR-155 [42], [43] and miR-1-2 [44] demonstrate that that these microRNAs are essential for normal immune function and cardiac development, respectively. Also, the miR-17/92 cluster has been knocked out in mice, resulting in a neonatal death characterized by with lung hypoplasia and a ventricular septal defect [45].

## Materials and Methods

### Mice and primary mouse embryonic fibroblasts (PMEFs)

129S1 and CzechII/Ei mice were purchased from Jackson laboratory and maintained according to the NIH animal care and use guidelines. All experiments involving animals were approved by the institutional review board. Androgenetic (AG) and parthenogenetic (PG) PMEFs were prepared as described previously [15]. For the current study, passage six PG PMEFs and passage seventeen AG PMEFs were utilized. For the imprinting analysis, PMEFs were generated from day 13 mouse embryos (day of plug = d1) from appropriate 129S1 and CzechII/Ei crosses. Embryos were dissected and washed twice in sterile phosphate buffered saline solution. Decapitated and eviscerated embryos were passed several times through an 18-gauge needle and resuspended on a per embryo basis in 1 ml 10% heat inactivated fetal bovine serum (FBS) in DMEM supplemented with 100 µg/ml DNase and 500 µg/ml collagenase. Triturated embryos were incubated at 37°C for thirty minutes, centrifuged at 1200 g for five minutes, and ultimately, plated with two disassociated embryos per a 150 mm dish in 10% FBS/DMEM with penicillin and streptomycin. PMEF cells were grown for two passages prior to RNA isolation.

### RNA Isolation and DNA microarray analysis

Total RNA was prepared from PMEF cells grown in a monolayer in 150 mm dishes using the Trizol reagent (Invitrogen) according to the manufacturer's directions. For DNA microarray analysis, mRNA was isolated from total RNA using the Oligotex mRNA purification system (Qiagen). Incyte Genomics reverse transcribed AG and PG polyA^+^ RNA with either Cy3 or Cy5 label. The labeled cDNA was then hybridized to the Incyte mouse GEM1 cDNA microarray according to their established protocol.

### Reverse transcription and polymerase chain reaction (RT-PCR)

Total RNA from PMEFs or mouse tissues was treated with RQ1 RNase-free DNase (Promega) to remove residual genomic DNA, followed by heat inactivation of the endonuclease. 5 µg RNA was reverse transcribed by Superscript II RNase H- RT (Invitrogen) in 60 µl reaction volumes according to the manufacturer's procedure, except that the reaction contained both 25 ng/µl Oligo dT and 5 ng/µl random hexamers. PCR was performed using 1 µl of the RT reaction as a starting material according to standard procedures. PCR cycling parameters were typically 4 minutes at 94°C, followed by thirty cycles of 30 sec at 94°C, 30 sec at 58°C, 90 sec at 72°C, and a final seven minute extension at 72°C. Supplemental Table S1 has the sequences of oligonucleotide primers used in this study.

### Imprinting analysis

RT-PCR products were generated from PMEF or brain RNA that was obtained from appropriate embryos or adult mice, respectively, derived from 129S1 and CzechII/Ei matings. Subsequently, RT-PCR products were cleaned using the MinElute PCR purification kit (Qiagen). RT-PCR samples were then treated with and without the indicated restriction enzymes obtained from New England Biolabs. Digests were analyzed by agarose gel electrophoresis with ethidium bromide staining. Supplemental Table S2 has 129S1 and CzechII/Ei restriction site polymorphisms discovered during the course of this study.

### cDNA Library screening and RNA Ligase Mediated-RACE (RLM-RACE)

An arrayed mouse day 19 embryo cDNA library (Origene MEA-1001) was screened by PCR to isolate *Irm*, *Meg8*, *Meg9*, *Peg11*, and *anti-Peg11* clones, while an adult mouse brain cDNA library was used for *Dlk1*/“DAT” cloning (Origene MAB-1001). The gene-specific primers for cDNA library screening are denoted by “#” in Supplemental Table S1. RLM-Race (Ambion) was performed for *Irm* according to the manufacturer's directions, with the initial PCR utilizing the 5′ RACE Outer and *Irm* 197Dn primers. The subsequent amplification used the same upstream primer and the *Irm* 120Dn primer.

### RNA expression analysis

Northern blots were purchased from Origene (MB-2020) and Clontech (7763-1). Blots were either hybridized with cDNA probes corresponding to the *Irm* sequence 19-555, *Dlk1* 830-1274), *Dlk1* Alt. pA (“DAT”) 1533-2044, *Meg3* 267-578, *Meg8* 1-294 (AF498299), or *Meg9* 2614-3009. Importantly, the *Irm* sequence that was selected contains sequences from exon 1 of *Rian*, thereby detecting both RNAs simultaneously. For *Peg11* and *Anti-Peg11*, three Northerns were performed. Specifically, a double stranded DNA probe corresponding to *Anti-Peg11* 477-1052 (Peg11 2977-3552) was used to detect both transcripts simultaneously. To distinguish signals derived from sense and antisense *Peg11*, two single stranded DNA probes corresponding to the same region were made and then used separately for Northerns. Blots were hybridized in ExpressHyb (Clontech) at 68°C for at least two hours. Blots were then washed twice in 2× SSC/0.1% SDS at room temperature for thirty minutes and twice in 0.1× SSC/0.1% SDS at 50°C for thirty minutes. Whole mount in situ hybridization was performed on dissected mouse embryos at different gestation stages, according to the conventional technique [46] with the following modifications: hybridization was performed at 68°C; an overnight wash at 68°C was added to limit non-specific RNA-RNA hybridization. To synthesize sense and antisense cRNA probes, a segment of *Irm* complimentary DNA spanning nucleotides 19-555 that contains part of the first *Rian* exon was labeled by DIG-UTP by run-off transcription (Roche).

## Results

### DNA microarray analysis of AG and PG PMEFs in the identification of imprinted genes

The identification of novel imprinted genes is often hampered by the difficulty in obtaining sufficient quantities of uniparental embryos or embryos with a particular uniparental disomy to screen for imprinted genes. To circumvent this problem, androgenetic (AG) and parthenogenetic (PG) PMEFs that maintain appropriate imprinted gene expression were established [15], [22]. Poly A+ RNA purified from both AG and PG PMEFs were subjected to RNA expression profiling using an Incyte mouse GEM1 array, containing 8,638 cDNA probes corresponding to identified genes [47]. In this screen, we expected to find both maternal and paternal imprinted genes to be differentially expressed. Genes that are modulated by passage number as well as genes whose expression depends on the levels of an imprinted gene product would also behave as differentially expressed. Table 1 shows the forty most differentially expressed genes as found by the DNA microarray analysis. Three known maternally expressed genes (*Grb10*, *Igf2r*, and *Meg3/Gtl2*) were identified, along with two paternally expressed transcripts (*Peg3* and *U2af35-rs1*), validating the experimental approach.

**Table 1:** Androgenetic versus Parthenogenetic PMEF Transcription Profiling Results Using Incyte GEM1 Microarray.

Differential Expression#	Gene Name	Accession Number	Chromosomal Location	Imprinted
−50.8	Periostin, osteoblast specific factor (*Postn*)	W81878*	3qC	
−26.2	Growth factor receptor bound protein 10 (*Grb10*)	AA260248*	11qAI	Yes
−13.5	Tenascin C (*Tnc*)	AA270625	4qC1	
−11.1	*Meg3/Gtl2* RNA	W97303*	12qF1	Yes
−9.7	5′-Methylthioadenosine phosphorylase (*Mtap*)	AA221942	4qC4	
−9.5	Thymus cell antigen 1, theta (*Thy1*)	W13151	9qA5.1	
−7.8	Imprinted RNA near Meg3 (*Irm*)	W89392*	12qF1	Yes (This study)
−6.2	Pleiotrophin (*Ptn*)	AA049699	6qB1	
−6.1	Calponin H1, smooth muscle (*Cnn1*)	W64636	9qA3	
−5.8	Cyclin-dependent kinase inhibitor 1A (P21) (*Cdkn1a*)	W88005	17qA3.3	
−5.5	Thrombospondin 2 (*Thbs2*)	AA003904	17qA2	
−4.8	Leukocyte immunoglobulin-like receptor subfamily B member 4 precursor (*Lilrb4*)	AA423373	10qB3	
−4.5	Insulin-like growth factor 2 receptor (*Igf2R*)	AA028475*	17qA1	Yes
−4.5	Dickkopf 3 (*Dkk-3*)	AA073904	7qF1	
−4.2	Phosphatidylinositol-binding clathrin assembly (*Picalm*)	AA139063	7qE1	
−4.2	Macrophage expressed gene 1 (*Mpeg1*)	AA268219*	19qA	
−4.0	Transcobalmin II (*Tcn2*)	AA220699	11qA1	
−4.0	A kinase (PRKA) anchor protein (gravin) 12 (*Akap12*)	AA403828	10qA1	
3.7	MAD2 (mitotic arrest deficient, homolog)-like 1 (*Mad2l1*)	AA002895	6qC1	
3.8	Kit ligand (*Kitl*)	AA403846	10qD1	
3.8	U2 small nuclear ribonucleoprotein auxiliary (*Zrsr1*)	AA274915*	11qA3.2	Yes
3.8	DNA primase small subunit, 49 kDa (*Prim1*)	AA259900	10qD3	
3.8	Cell division cycle 2 homolog A (*Cdc2a*)	AA035888	10qB5.3	
4.0	Minichromosome maintenance complex component 4 (*Mcm4*)	AA259788	16qA2	
4.0	Cyclin B1 (*Ccnb1*)	AA396324	13qD1	
4.2	Aldehyde dehydrogenase family 1, subfamily A7 (*Aldh1a7*)	AA122814	19qB	
4.2	H2A histone family, member Z (*H2afz*)	AA466087	3qG3	
4.2	EST (ETn)	AA184421	Multiple	
4.3	Paternally expressed gene 3 (*Peg3*)	AA003064*	7qA1	Yes
4.3	Minichromosome maintenance deficient 5 (*Mcm5*)	AA031056	8qC1	
4.4	AA125385	AA125385	11qE2	
4.4	Cyclin B1 (*Ccnb1*)	AA124592	13qD1	
4.4	Rac GTPase activating protein 1 (*Racgap1*)	AA140523*	15qF1	
4.6	EST (ETn)	AA105996	Multiple	
4.9	Aquaporin 1 (*Aqp1*)	AA241281	6qB3	
5.1	EST (ETn)	AI608121	Multiple	
5.2	Public domain EST (MuLV gag, pol, env)	AA087673	Multiple	
6.8	Hematopoietic progenitor cell antigen CD34 (*CD34*)	AA064307	1qH6	
8.3	Solute carrier family 9 (*sodium*/hydrogen exchanger), member 3 regulator 1 (*Slc9a3r1*)	AA239009	11qE2	
9.6	Neuronal membrane glycoprotein M6-a (*Gpm6a*)	AA269845	8qB1.3	

# Represents fold change of higher signal/lower signal (Negative numbers are for ESTs higher in parthenogenetic PMEFs, while positive numbers for those higher in androgenetic PMEFs).

### Identification of *Irm* as a candidate maternally expressed gene in mice

**Figure 1:**
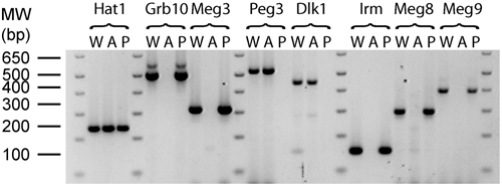
Androgenetic and Parthenogenetic PMEFs maintain appropriate imprinted gene expression. RT-PCR was performed on indicated PMEF cell lines: W (Wild-type), A (Androgenetic), and P (Parthenogenetic). Histone acetyltransferase 1 (*Hat1*) is a biallelically expressed control. *Dlk1* and *Peg3* are controls for paternal expression while *Grb10* and *Meg3/Gtl2* are controls for maternal expression. With *Irm* primers 1307Up and 1581Dn, *Irm* RNA expression was undetectable in AG PMEF, while its expression was abundant in WT and PG PMEFs. Moreover, *Meg8* and *Meg9*, two other genes in the large imprinted gene cluster that contains *Irm*, have expression patterns that are consistent with being imprinted with maternal expression. The PCR primers were *Hat1* 510Up/758Dn, *Grb10* 270Up/770Dn, *Meg3* 267Up/578Dn, *Peg3* 4430Up/4956Dn, *Dlk1* 830Up/1274Dn, *Irm* 988Up/1140Dn, *Meg8* 1Up/294Dn (AF498299) and *Meg9* 2614-3009.

**Figure 2:**
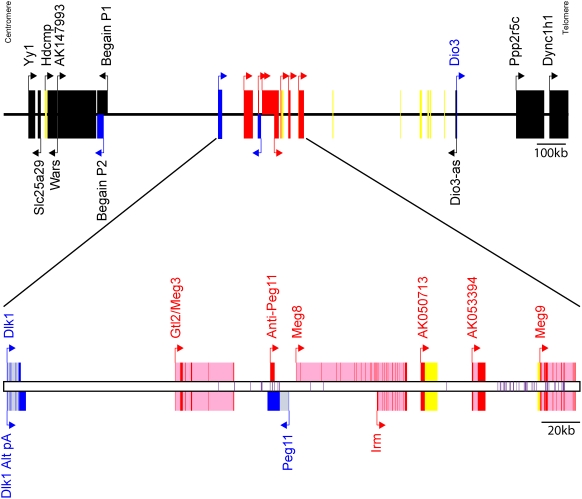
Genomic Organization of *Dlk1-Dio3* Imprinted Region. This figure summarizes our results about the imprinting and structure of genes in this mouse region. Arrows denote transcriptional orientations. Genes written in black were shown to be biallelically expressed. Genes drawn in blue are paternally expressed, while those in red are maternally expressed. Genes written in yellow were undetectable by RT-PCR. For *Begain* transcribed from promoter 2, this transcript was coded blue as it is paternally expressed in sheep in a tissue specific manner, although in this study, RT-PCR failed to amplify a transcript derived from this promoter. Of these, accession numbers, AK141557, AK163826, AK048151, and AK044800, correspond to unspliced “transcripts” whose cDNA sequence ends in a polyA stretch of genomic DNA, suggesting that these “cDNAs” may be genomic DNA contamination in the Riken mouse cDNA libraries. For the bottom panel, exons are dark colored while introns are light. Purple lines represent the position of microRNA precursors. In all, fifty-two mouse microRNA precursors as listed in Table 2 map to this imprinted region.

For each of the forty ESTs that are listed in Table 1, RT-PCR was performed on total RNA from wild-type (WT), AG, and PG PMEFs to determine if expression was consistent with being imprinted. Figure 1 shows a typical RT-PCR result and demonstrates that appropriate imprinted gene expression is maintained in these uniparental cell lines. *Grb10*, *Meg3/Gtl2*, *Peg3*, and *Dlk1* were selected as imprinted gene controls. As expected, the biallelic gene histone acetyltransferase 1 (*Hat1*) and imprinted genes (*Grb10*, *Meg3/Gtl2*, *Peg3* and *Dlk1*) showed appropriate expression by RT-PCR in the three cell lines (Figure 1). Ultimately, nine of the forty ESTs (denoted by stars in Table 1) were highly differentially expressed between AG and PG PMEFs by RT-PCR (data not shown). Of these nine, five ESTs corresponded to imprinted genes that were known at the time (*Grb10*, *Igf2r*, *Meg3/Gtl2*, *Peg3*, and *U2af35-rs1*). This result suggested the possibility that a subset of the four remaining ESTs (corresponding to *Postn*, *Mpeg1*, *Racgap1*, and *Irm*) may in fact be imprinted. To demonstrate conclusively the imprinting status of a gene, an assessment of allele specific transcription was performed using an informative polymorphism for the four ESTs whose imprinting status was unknown. We found that *Postn*, *Mpeg1*, and *Racgap1* were biallelically expressed (data not shown), suggesting that the genes corresponding to these ESTs may be regulated by passage number or by an imprinted gene product. Intriguingly, the final EST W89392 (*Irm*) with its high RNA levels in PG versus AG PMEFs connected to other ESTs that mapped to the same BAC (AC040981.2) as the known mouse imprinted gene *Meg3/Gtl2*. *Meg3/Gtl2* is the founding member of a much larger imprinted gene region that has been implicated as having parent of origin effects in three mammalian species. Hence, an exhaustive screen was undertaken for a 1.96 Mb region around *Irm* to investigate the imprinting status of all contained mRNAs. For the sake of clarity, results will be presented from centromere to telomere. Figure 2 shows the genomic organization of this region in mice.

### Defining the centromeric and telomeric ends of the imprinted gene cluster in PMEFs

Six mRNAs (*Yy1*, *Slc25a29*, *Hdcmp*, *Wars*, *AK147993*, and *Begain*) as depicted in Figure 2 that are on the centromeric side of *Dlk1* were tested by RT-PCR in WT, AG, and PG PMEFs to determine if they were differentially expressed between these samples. Similarly, two mRNAs, *Ppp2r5c* and *Dync1h1*, on the telomeric side of *Dio3* were also tested. For seven of the eight genes, RT-PCR products were obtained from the three cell lines at similar levels, indicating that these genes are biallelically expressed in PMEFs (data not shown). Mouse *Hdcmp* (accession number AI132487) was undetectable by RT-PCR. Since the neighboring genes, *Yy1*, *Slc25a29*, *Wars*, and *AK147993*, are not imprinted in PMEFs, it is unlikely that *Hdcmp* is imprinted (see Figure 2).

For *Begain* RT-PCR, our original primer set fell within the region that is common to *Begain* mRNA irrespective of which of the two mouse *Begain* promoters is used. Our results were consistent with biallelic expression. In sheep, *Begain* has been recently shown to be biallelically expressed in all tissues when transcription is initiated at one of its two promoters [48]. Transcripts derived from the other promoter in sheep are characterized by imprinted expression in a tissue specific manner. Since our RT-PCR product would detect transcripts derived from both promoters, multiple primer sets were designed to investigate expression from each mouse promoter. Unfortunately, the alternative first exons of *Begain* are quite GC rich and specific amplifications products were not observed. Hence, we can only conclude that at least one of the two *Begain* promoters is biallelic.

### Imprinting, Expression, and cDNA Cloning of *Dlk1* and Its Alternative Polyadenylated Transcript “*DAT*”

**Figure 3:**
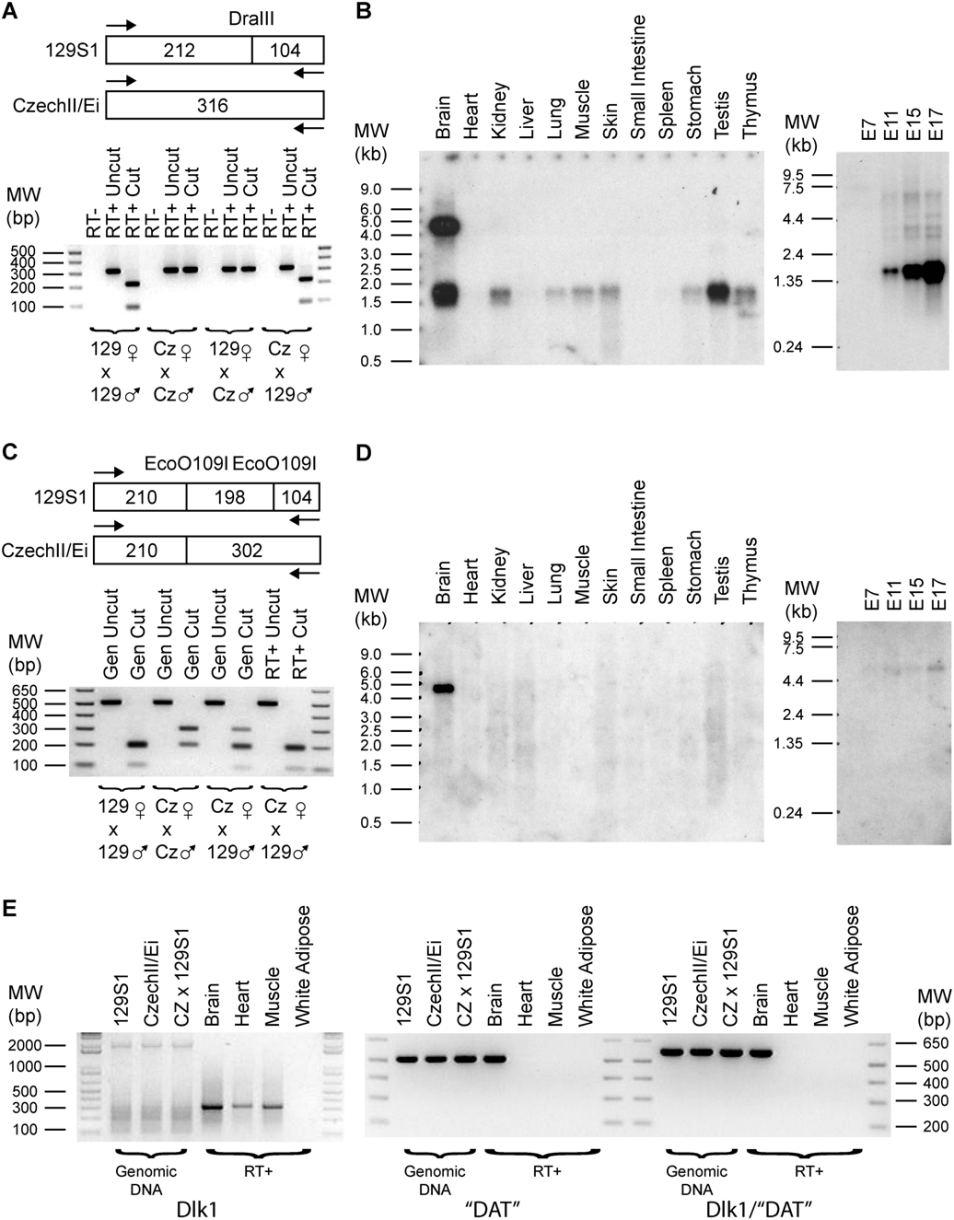
*Dlk1* is paternally expressed and has an abundant alternatively polyadenylated transcript in the mouse brain. (A) A DraIII restriction site polymorphism between 129S1 and CzechII/Ei mice was utilized to determine that *Dlk1* is paternally expressed. PCR primers were *Dlk1* 2Up/317Dn. (B) Northern analysis using the same *Dlk1* PCR fragment as probe revealed that *Dlk1* is widely expressed. Of the tissues investigated, only the adult brain is characterized by having an additional and abundant transcript that is roughly 4.5 kb in length. (C) Several ESTs cluster to the syntenic region of “DAT” (*Dlk1* associated transcript). RT-PCR was done with “DAT” primers 1533Up/2044Dn. Using a EcoO109I restriction site polymorphism, “DAT” was shown to be like *Dlk1* in its paternal expression. (D) Northern analysis on adult tissue samples of “DAT” detected a 4.5 kb transcript only in the brain. This band is identical in size to the long transcript of *Dlk1* found in the brain. Moreover, the length of this transcript is greater than the distance (∼2.7 kb) between mouse *Dlk1* polyA and “DAT” polyA. (E) RT-PCR was performed with various primer sets. Left most panel showed that *Dlk1* is expressed in brain, heart and muscle. Middle panel shows that “DAT” is only detectable in brain and is consistent with Northern findings. For the right panel, PCR was performed with a primer set in which the upstream primer (*Dlk1* 1237Up) was before the canonical *Dlk1* polyA site while the downstream primer was after this site (*Dlk1* 1805Dn). A band was detected only in the brain, indicating that “DAT” is an alternatively polyadenylated transcript of *Dlk1*. This result was confirmed by cDNA cloning (Accession numbers: EU434914-EU434917).

*Dlk1* is paternally expressed in mice [25], [27] and in other mammalian species with the exception of marsupials [26], [49]. A DraIII restriction site polymorphism between CzechII/Ei and 129S1 mice in the *Dlk1* cDNA was identified and used to confirm the imprinted status of *Dlk1* (Figure 3A). Northern analysis showed that *Dlk1* is expressed in many adult mouse tissues, including brain, kidney, testis, and thymus. Mouse brain expressed the most *Dlk1* mRNA and is characterized by an additional and abundant 4.5 kb transcript that was not detected in the other investigated tissues (Figure 3B).

In sheep and humans, a neighboring gene “DAT” (DLK1 Associated Transcript) is also paternally expressed [23]. In addition to their common paternal expression, DLK1 and “DAT” lie in the same transcriptional orientation with the polyA of DLK1 roughly 3.1 kb upstream of the polyA of “DAT” in humans. In mice, several ESTs from Unigene cluster Mm.458824 map to the syntenic region of “DAT”. An EcoO109I restriction site polymorphism demonstrated that “DAT” is paternally expressed in mice (Figure 3C). Northern analysis detected “DAT” only in the brain and its 4.5 kb length was identical to the brain-specific long form of mouse *Dlk1* (Figure 3D). This finding in conjunction with the fact that the genomic distance between the polyAs of *Dlk1* and “DAT” in mice is ∼2.7 kb indicated that “DAT” was most likely an alternatively polyadenylated transcript derived from the *Dlk1* gene. To test this hypothesis, RT-PCR was performed with primers before and after the canonical *Dlk1* polyA site and yielded a product only in mouse brain (Figure 3E). Moreover, four independent cDNA clones from the Origene mouse brain cDNA library (MAB-1001) were isolated and sequenced, demonstrating unequivocally that the “DAT” sequences result from an alternative polyadenylated product from the *Dlk1* gene (Accession numbers: EU434914-EU434917). The lengths of our two longest clones that contain the entire *Dlk1* ORF are consistent with the size of the 4.5 kb form of alternatively polyadenylated *Dlk1*. The significance of this alternative pre-mRNA processing event is unknown, but EST analysis indicates that is conserved across mammals (data not shown). In addition to alternative polyadenylation, our isolated cDNA clones are characterized by alternative splicing within the *Dlk1* ORF. The existence of these alternatively spliced products was independently confirmed by several mouse *Dlk1* mRNAs and ESTs in GenBank.

### *Meg3/Gtl2*: Imprinting, Expression, and miR-770

**Figure 4:**
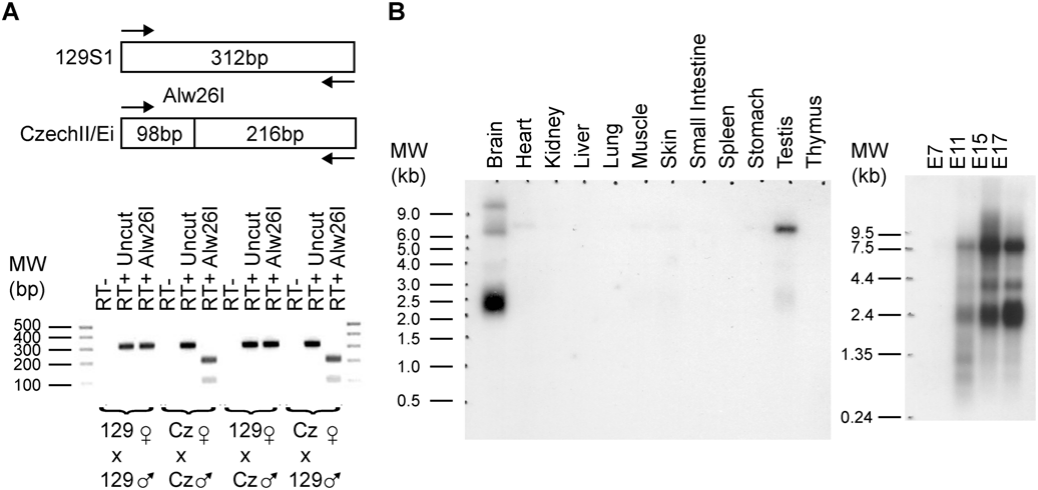
*Meg3* is maternally expressed and highly expressed in the brain and testis. (A) Alw26I restriction digests on RT-PCR products confirm that *Meg3* is maternally expressed. For PCR, *Meg3* 267Up/578Dn primers were used. (B) Northern blots reveal that *Meg3* is characterized by alternative splicing. In the adult mouse brain, the predominant band is roughly 2.4 kb in length while in testis is 6.5 kb.

Figure 4A shows that *Meg3/Gtl2* is maternally expressed using an identified Alw26I restriction site polymorphism between CzechII/Ei and 129S1 mice. This result confirms several earlier studies [24], [50]. By Northern analysis, *Meg3/Gtl2* is highly expressed in the brain and testis (see Figure 4B). In adult mouse brain, the predominant form is 2.4 kb, while in testis, a 6.5 kb transcript is most abundant. In addition, *Meg3/Gtl2* contains in its last intron (Relative to Accession number Y13832) the evolutionarily conserved microRNA miR-770 (see Figure 2 and Table 2). This observation is intriguing since *H19*, a maternally expressed noncoding RNA near *Igf2*, also is characterized by having an evolutionarily conserved microRNA miR-675 [51]. Table 2 shows several mRNAs that include *Bmp1*, *Bmp15*, *Fosb*, *Lmna*, *Sspn*, *Tmod1*, and *Trp53* that are predicted to be regulated by miR-770. If these genes are *bona fide* targets, loss of miR-770 that occurs in the associated pUPD syndromes could contribute to the observed phenotypes by increasing the levels of its targeted genes, many of which are implicated in placental, bone, and muscle biology.

**Table 2:** Mouse MicroRNAs located in Dlk1-Dio3 Imprinted Region.

Mouse MicroRNA Host Gene	Associated MicroRNAs	Example of Mouse mRNAs predicted to be microRNA targets according to miRanda for given microRNA (http://www.microrna.org/microrna/)
Meg3	mmu-miR-770 #	Bmp1, Bmp15, Capn3, Casq2, Fosb, Lmna, Mb, Obscn, Peg10, Ppp1ca, Sspn, Tmod1, **Trp53**
Unidentified	mmu-miR-673	Camk2a, Camk2b, Camk2d, Camk2g, Dnmt1, Mtpn, Myh6, Ndn, Pax3, Rbl1, Sln, Tnnt1, Wnt1
Unidentified	mmu-miR-493 #	Cacng5, Camk2g, Cdkn1c, Ctcf, Dag1, Fhl1, Fos, Hras1, Jun, Mib2, Mtap, Peg10, Shh, Tmod1
Unidentified	mmu-miR-337 #	Capza2, Des, Dmd, Dnmt3a, Myh8, Mypn, Nfatc1, Plagl2, Pvalb, Sgcb, Snta1, Tpm3, **Trp53**
Unidentified	mmu-miR-540	Akt3, Bmp2, Bmp7, Capzb, Emd, Itga7, Itgb1, Msc, Myog, Nkx2-5, Pten, Rhoa, Sln, Tlx1, Vim
Unidentified	mmu-miR-665 #	Casq2, Igf2, Junb, Ldb3, Peg10, Magel2, Nnat, Pax3, Ryr1, Sntb2, Tln1, Tpm2, **Trp53**, Ttn
Anti-Peg11 $	mmu-miR-431 # d	Camk2b, Casq1, Dtna, E2f1, Fgf4, Gata3, Igf1, Kit, Max, Peg10, Plagl2, Ppp3r1, Sgcd, Tcf21
Anti-Peg11 $	mmu-miR-433 #	Bmpr1b, Capza1, Creb1, Ctcf, E2f3, Gata6, Isl1, Jak2, Myh9, Peg10, Plagl2, Ppp3r1, Sntg1
Anti-Peg11 $	mmu-miR-127 # d e	Auts2, Bcl6, Camk2d, Cdc42, Creb5, E2f3, Igf2, Myo1c, Otx1, Plagl2, Pitx2. Sp4, Sspn, Xpo5
Anti-Peg11 $	mmu-miR-434	Atp2a2, Bmp2, Cacng5, Calm1, Calm3, Camk2g, Dtna, Fhl1, Lmnb1, Nfatc2, Pdlim2, Plagl2
Anti-Peg11 $	mmu-miR-136 # d	Ankrd1, Brca1, Calm2, Dmd, Lmna, Myoz1, Plagl1, Pln, Sgcd, Sgce, Syne1, Tcap, **Trp53**
Meg8	mmu-miR-341	Atp2a2, Camk2b, Grb10, Hoxb8, Igf2bp2, Itgb1bp2, Map2k6, Nebl, Rab5c, Satb2, Sgcz, Tpm3
Meg8	mmu-miR-1188	Predicted targets are not currently available
Meg8	mmu-miR-370 #	Cacng5, Capza1, Casq2, Itgb1bp2, Lmna, Nbr1, Peg10, Ppp3ca, Tln1, Tpm4, **Trp53**, Zyx
Unidentified	mmu-miR-882 +	Acta1, Dmn, Lepr, Mecp2, Myh7b, Myh8, Myl3, Myo9b, Myoc, Nes, Pdlim1, Sntg2, Tmod4, Vcl
Unidentified	mmu-miR-379 #	Bmp2, Capza2, Cdk2, Cdk8, Cdkn1a, Ctcf, Dkk3, Dmd, Dnmt3b, Jun, Mypn, Ppp3r1, Sntg1
Unidentified	mmu-miR-411 #	Camk2g, Ckmt2, Dmd, Ldb3, Lmnb2, Map2k1, Myoz2, Pten, Runx2, Sgcz, Tpm1, **Trp53**, Ttn
Unidentified	mmu-miR-299 #	Aqp1, Asb4, Bmpr1a, Dkk3, Dtna, Fgf1, Igf1, Igf2, Kit, Myh9, Myot, Nfatc2, Rb1, Sp4, Sync
Unidentified	mmu-miR-380 #	Bcl6, Bmp4, Bmp15, Bmpr1a, Bmpr1b, Hic1, Pdlim3, Peg10, Pitx2, Pln, Rb1, Sgcd, Sln, Tcap
Unidentified	mmu-miR-1197 #	Predicted targets are not currently available
Unidentified	mmu-miR-323 #	Ankrd23, Camk2a, Camk2d, Camk2g, Capza1, Capzb, Fgf10, Flnc, Ldb3, Myh13, Pax7, Pdlim2
Unidentified	mmu-miR-758 #	Bmp7, Cacng5, Capza2, Dmn, Dtna, Emd, Itgb1, Lmnb1, Peg10, Rb1, Sgcb, Sntb2, Tpm4
Unidentified	mmu-miR-329 #	Camk2a, Capza1, Capzb, Dmd, Fez2, Fgf10, Igf2, Ldb3, Mycs, Mypn, Peg10, Sspn, Sync
Unidentified	mmu-miR-494 #	Bmp15, Camk2a, Camk2d, Fhl1, Nbr1, Pax3, Peg10, Pitx2, Plagl1, Rb1, Syne1, Utrn, Vcl
Unidentified	mmu-miR-679 +	Capzb, **Dlk1**, Myh11, Myh7b, Myl6b, Pdcd4, Pitx2, Sgca, Smpx, Synpo2, Trp53bp1, Wwox
Unidentified	mmu-miR-1193	Predicted targets are not currently available
Unidentified	mmu-miR-666	Predicted targets are not currently available
Unidentified	mmu-miR-543 #	Calm3, Camk2a, Camk2g, Ctcf, Dmn, Lmna, Mycn, Myot, Nbr1, Peg10, Rb1, Rbl1, Sntb2
Unidentified	mmu-miR-495 #	Atp2a2, Bmp2, Calm3, Camk2g, Capza1, Dag1, Isl1, Peg10, Pvalb, Rb1, Sgcz, Sntg1, Tpm3
Unidentified	mmu-miR-667 +	Ankrd1, Cacng1, Camk2b, Des, Emd, Flnb, Myh14, Myh7b, Pdlim3, Ppargc1a, Sgcg, Tcap, Utrn
Unidentified	mmu-miR-376c #	Cacng5, Dag1, **Dlk1**, Lmnb1, Mycn, Nbr1, Nfatc1, Nfatc2, Plagl2, Ppp3r1, Sgcd, Syne1, Tpm4
Unidentified	mmu-miR-654 #	Ankrd23, Cdkn1c, Cryab, Fos, Igf2r, Jun, Lmna, Myl3, Myom2, Plagl1, Sgcb, Sntb2, Ybx1
Unidentified	mmu-miR-376b #	Camk2b, Camk2g, Capza1, Ctcf, Dmd, Emd, Hmga2, Igf1, Myc, Peg10, Plagl2, Pml, Ppp3r1
Unidentified	mmu-miR-376a #	Atp2a2, Bach1, Bmp2, **Dlk1**, Fgf14, Kit, Mest, Peg12, Ppp3r1, Sema3a, Socs6, Trp63, Wnt3a
Unidentified	mmu-miR-300 #	Bcl2, Bcl6, Bmp2, Camk2a, Camk2b, Dmd, Dmn, Emd, Fgf10, Fhl1, Isl1, Ldb3, Mycn, Tcf21
Unidentified	mmu-miR-381 #	Auts2, Camk2a, Camk2g, Capza2, Emd, Grb10, Ldb3, Lmna, Nbr1, Pax3, Pax7, Plagl1, Sntg1
Unidentified	mmu-miR-487b #	Asb4, Ctcf, Dio2, Dtna, Fgf5, Fgfr3, Fhl1, Foxp2, Pitx2, Ptpro, Runx2, Spock2, Sumo2, Vegfa
Unidentified	mmu-miR-539 #	Brca1, Bmp5, Bmpr1a, Bmpr1b, Camk2d, Casq1, Ctcf, Flnc, H2afz, Itgb1bp2, Myf5, Rbl2, Rhoa
Unidentified	mmu-miR-544 #	Bmpr2, Calm1, Calm3, Cdkn1c, **Dlk1**, Lmnb1, Myh1, Peg3, Pln, Rb1, Sgcz, Sntb2, Tpm1, Xpo5
Meg9	mmu-miR-382 #	Atp2a2, Calm2, Camk2g, Dag1, Fgf10, Kras, Myf5, Myot, Mypn, Plagl1,Pdlim5, Sgce, Ttn, Vim
Meg9	mmu-miR-134 # f	Bmp1, Bmp2k, Camk2d, Ctcf, Dmd, Emd, Itgb1, Mycn, Obscn, Plagl1, Ppp3r1, Ryr1, Tcf21
Meg9	mmu-miR-668 #	Calm1, Camk2a, Camk2g, **Dlk1**, Dmn, Flnc, Myog, Pdlim2, Peg10, Sgcd, Sspn, Tcf21, Tlx1
Meg9	mmu-miR-485 #	Actg2, Capza1, Dag1, Isl1, Lmna, Myl2, Pax3, Rhoa, Ryr1, Sntb1, Tpm3, Tpm4, **Trp53**, Zyx
Meg9	mmu-miR-453 # +	Cdk4, Fgf3, Fgfr1, H19, Myh11, Myl7, Myoc, Pon3, Ryr1, Sgce, Sin3a, Sntb1, Tcn2, Tnnc2
Meg9	mmu-miR-154 #	Ankrd23, Bmp2, Bmp5, Bmpr1a, Bmpr1b, Capza2, Ctcf, Grb10, Hras1, Igf1, Igf2, Pax3, Plagl1
Meg9	mmu-miR-496 #	Atp2a2, Bcl2, Bmp15, Calm1, Capza1, Dmd, Dtna, Kras, Mest, Rb1, Sgcb, Sln, Sntg1, Vcl
Meg9	mmu-miR-377 #	Cacng5, Capzb, Ctcf, **Dlk1**, Emd, Flnc, Nfatc2, Pitx2, Plagl2, Rb1, Sgcd, Sntg2, Syne1, Tcap
Meg9	mmu-miR-541 #	Actn2, Ankrd23, Bmp2, Ckmt2, Capn3, Fosb, Pax7, Pitx2, Sgcz, Sln, Sspn, Tmod4, Tpm4
Meg9	mmu-miR-409 #	Actg2, Calm3, Camk2a, Camk2g, Ctcf, Dmd, Nfatc1, Obscn, Ppargc1a, Ryr1, Tmod1, Tpm1
Meg9	mmu-miR-412 #	Bach1, Bach2, Bard1, Bcl2, Bmp2k, Camk2b, Dach1, Dtna, Fhl1, Igf2, Myl2, Pdlim2, Peg10, Ttn
Meg9	mmu-miR-369 #	Atp2a2, Capza2, Dicer1, Dmd, Dmn, Fgf10, Fhit, Ldb3, Lmnb1, Nbr1, Ppp3r1, Sgcb, Tln1, Utrn
Meg9	mmu-miR-410 #	Ankrd1, Bcl11a, Bmp2k, Bmpr1a, Bmpr1b, Ctcf, Dido1, Dtna, Foxp2, Hgf, Nfatc2, Otx2, Pvalb

# MicroRNA is also identified in human.

d Previously shown to regulate negatively mouse *Peg11* (*Rtl1*) expression [52].

e Previously shown to regulate Bcl6 [53].

f Previously shown to regulate negatively mouse Limk1 [61], Nanog, and LRH1 [62].

$ Maps to Anti-Peg11 region although it should be noted that the ends of this transcript have not been defined.

+ Predictions from http://microrna.sanger.ac.uk/targets/v5/, since microRNA targets were absent from miRanda website.

### Peg11 and Anti-Peg11: Imprinting, Expression, cDNA Cloning, and MicroRNAs

**Figure 5:**
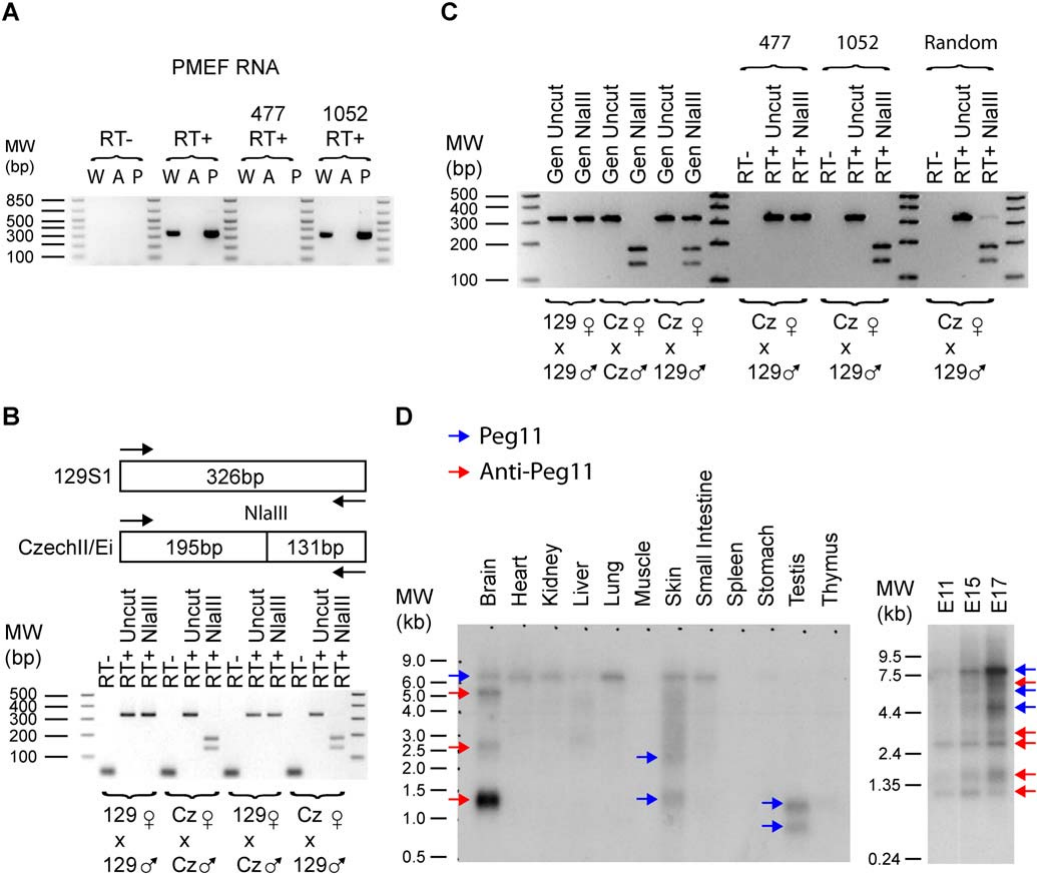
Imprinting and expression analysis of mouse *Peg11* and *anti-Peg11*. (A) RT-PCR was performed on RNA derived from wild-type {W}, Androgenetic {A} and Parthenogenetic {P} PMEF cell lines. RT reactions were performed with either random hexamers or the strand-specific primers for *Peg11* “477” or *Anti-Peg11* “1052”. For PCR, Anti-Peg11 522Up/847Dn primers were used. These results demonstrate that in PMEFs *Anti-Peg11* is expressed but not *Peg11*. (B) An NlaIII restriction site polymorphism between 129S1 and CzechII/Ei was utilized to demonstrate that *Anti-Peg11* is maternally expressed in PMEFs. (C) As in panel A, three RT reactions were performed with the same PCR primers but on adult brain RNA derived from a mouse with a CzechII/Ei mother and 129S1 father. These results demonstrate that *Anti-Peg11* and *Peg11* are both expressed, although *Anti-Peg11* is more abundant in mouse brain. *Peg11* was paternally expressed, while *Anti-Peg11* was maternally expressed. PCR on genomic DNA (Gen) revealed that the primers did not preferentially amplify either the 129S1 or CzechII/Ei alleles. (D) Northern for *Peg11* and *Anti-Peg11* using a double stranded DNA probe corresponding to *Anti-Peg11* 477-1052. Blue arrows indicate the position of *Peg11* transcripts, while red arrows correspond to *Anti-Peg11*. This annotation was possible as the Northern was repeated with single stranded probes for both *Peg11* and *Anti-Peg11* (data not shown).

Unlike other genes in this region where oligo(dT) and random primed reverse transcription reactions were used as a substrate for PCR, we also performed strand-specific RT reactions to ascertain if the observed product corresponded to *Peg11* or its antisense transcript. As Figure 5A–C demonstrates, *Anti-Peg11* was expressed from the maternal allele in both PMEFs and adult brain. In contrast, *Peg11* was undetectable in PMEFs and was expressed at lower levels in the brain in comparison to its antisense transcript. Our results showed that *Peg11* was paternally expressed in brain. Northern analyses were performed with double stranded DNA probes and then sequentially with two single-stranded probes. Figure 5D shows that *Peg11* is broadly expressed but at low levels in many tissues. *Anti-Peg11* on the other hand is far more restricted in its expression with a prominent signal only detected in the brain.

To date, there are no reports of a cloned cDNA corresponding to either *Peg11* or *Anti-Peg11* in any species. In mice, a computationally predicted open reading frame (ORF) termed Retrotransposon-like 1 (*Rtl1*) has been deposited in GenBank (NM_184109) that maps to *Peg11*. Hence, an arrayed day 19 embryonic cDNA library (Origene MEA-1001) was screened by PCR with *Anti-Peg11* 477Up and 1052Dn primer pair. This primer set does not distinguish between strands and would yield a product for both *Peg11* and *Anti-Peg11*. Ultimately, two *Peg11* cDNAs were isolated and sequenced. Both clones (Accession numbers: EU434914 and EU434918) exceeded 6 kb in length, revealing that *Peg11* has two exons (see Figure 2). Given the length of the *Peg11* as determined by Northern, our cDNA clones are nearly full length. Likewise, a 2.3 kb *Anti-Peg11* cDNA was isolated and sequenced (Accession number EU434921). As Figure 2 shows, a large number of microRNAs map to the *Anti-Peg11/Peg11* region and all are in the same transcriptional orientation as *Anti-Peg11*. Our *Anti-Peg11* cDNA clone most likely represent a cleavage product of a longer *Anti-Peg11* message that resulted as the primary miRNA transcript is processed into pre-miRNAs. This conclusion is based on the observation that our cDNA begins at the end of miR-431 precursor and ends at the beginning of miR-127 precursor. Multiple experiments were unsuccessful in isolating cDNA clones that were upstream or downstream of our cloned sequence. Since *Anti-Peg11* serves as a primary microRNA transcript, it would be nearly impossible to detect a full length *Anti-Peg11* under conditions where the Microprocessor complex is functioning.

A large number of microRNAs map to the *Anti-Peg11/Peg11* region (see Figure 2 and Table 2). Three microRNAs in this region, miR-431, miR-127, and miR-136, were shown previously to regulate *Peg11* through a siRNA-like mechanism [52]. Also, miR-127 was shown to regulate *Bcl6* [53].Since individual microRNAs regulate large sets of genes often numbering in the hundreds [40], [41], we investigated the predicted targets for all microRNAs in this region. As Table 2 shows, several predicted targets for microRNAs in this region have been implicated in processes that when perturbed could result in phenotypes associated with the human and mouse UPD syndromes. These genes include *Bmp2*, *Casq2*, *E2f1*, *E2f3*, *Grb10*, *Igf2*, *Jak2*, *Junb*, *Ldb3*, *Peg10*, *Plagl2*, *Magel2*, *Nnat*, *Pax3*, *Ryr1*, *Sgce*, *Sntb2*, *Sspn*, *Tcf21*, *Tln1*, *Tpm2*, *Trp53*, *Ttn*, and *Xpo5*.

### *Meg8*: Imprinting, Expression, cDNA Cloning and MicroRNAs

**Figure 6:**
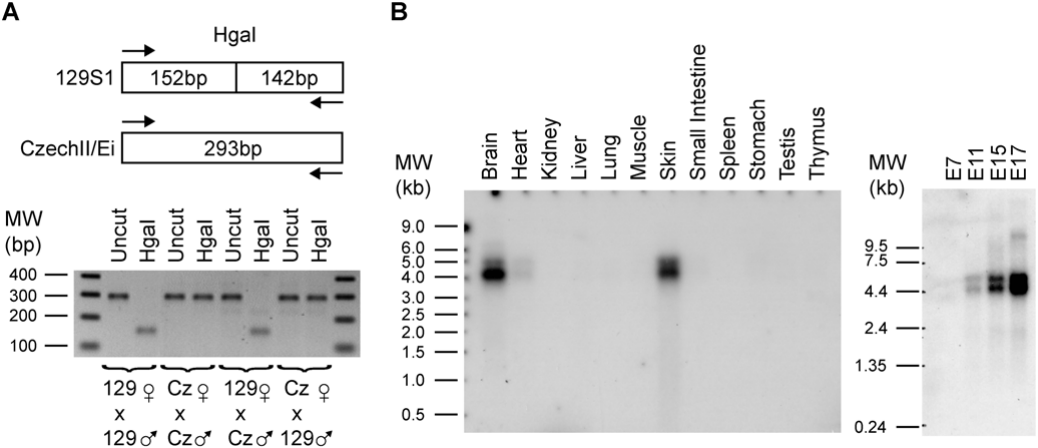
*Meg8* is expressed from the maternal allele and highly in the brain and skin. (A) The *Meg8* alleles contain an informative restriction site polymorphism in that a HgaI site is present in the 129S1 allele in comparison to CzechII/Ei. RT-PCR products with AF498299 1Up/294Dn primers for *Meg8* from the four intraspecific crosses were digested with and without HgaI, verifying the imprinted expression of this gene. (B) Northern blots were performed on adult tissues (left) and embryos harvested at different time points (right). *Meg8* levels were highest in brain and testis. In addition, *Meg8* RNA increases as a functional of embryonic development.

**Figure 7:**
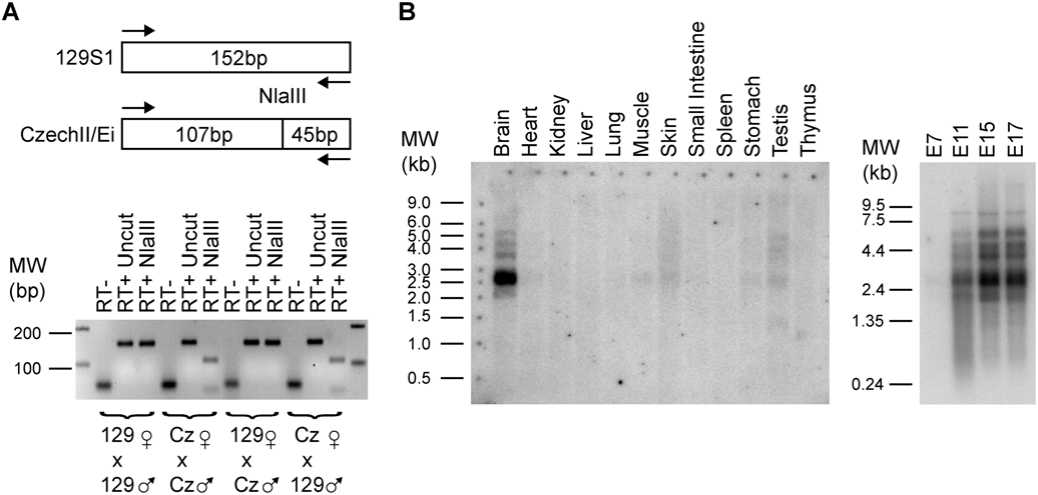
*Irm* is maternally expressed, alternatively spliced, and abundant in the adult brain. (A). A single nucleotide polymorphism in *Irm* cDNA was identified at 1094A→T between 129S1 and CzechII/Ei mouse strains that creates a NlaIII restriction site in the CzechII/Ei *Irm* cDNA. RT-PCR was performed with *Irm* primers 988Up and 1140Dn. Restriction digests of RT-PCR products from intraspecific crosses between these two subspecies by NlaIII demonstrated that *Irm* is expressed in a monoallelic manner from the maternal allele. (B) An adult tissue (left) and total embryo from different gestational days (right) polyA^+^ RNA blot (left) was hybridized with a *Irm/Rian* exon 1 cDNA probe. A 2.5 kb transcript was predominantly detected in the brain and to a significantly lesser extent in testis, stomach, and muscle. Higher molecular weight bands of lower relative abundance in comparison to the 2.5 kb message were also seen that reflect the heterogeneity in spliced variants. Since *Rian* RNA is approximately 5.4 kb, the predominant RNA product from the *Irm/Rian* promoter is *Irm* RNA, while *Rian* accounts for at most 5% of total transcripts. This result is consistent with the relative abundance of ESTs that are specific for each of the alternatively spliced products.

As Figure 6A demonstrates, *Meg8* is a maternally expressed gene in mice as deduced from digests of RT-PCR products using an informative HgaI restriction site polymorphism between 129S1 and Czech II/Ei mice. To date, no reports have documented the cloning of *Meg8* in any mammalian species. As such, the Origene arrayed day 19 cDNA library (MEA-1001) was screened for a full length *Meg8* cDNA. The sequence of the isolated *Meg8* cDNA has been deposited in GenBank (Accession number: EU434919). Comparison of *Meg8* cDNA with the mouse genome revealed that this transcript has 26 exons (Figure 2). Conceptual translation of *Meg8* revealed that it is a noncoding RNA that lacks significant ORFs (data not shown). Importantly, we found that *Meg8* and *Irm* share ten common exons. Northern analysis for the region using a probe that is unique to *Meg8* cDNA showed that it is expressed highly in the adult mouse brain and skin (Figure 6B). Significantly lower levels of *Meg8* were detected in the heart. In contrast, *Irm* is barely detectable in the skin (Figure 7B), demonstrating that *Meg8* and *Irm* differ in their expression patterns. Moreover, only *Meg8* transcripts have the intron-encoded microRNAs miR-341, miR-1188, and miR-370 that lie upstream of *Irm* (see Figure 2 and Table 2). Of note, miR-370 is also conserved in humans. Several genes that are predicted to be regulated by these microRNAs are shown in Table 2 and include *Grb10*, *Lmna*, *Peg10*, and *Trp53*.

### *Irm*: Imprinting, Expression, RLM-RACE and cDNA cloning

From the original large scale transcription profiling of AG and PG PMEFs, EST W89392 was an attractive candidate for a novel imprinted transcript. Since no polymorphisms were detected within this EST, a cDNA library derived from mouse day 19 embryos (Origene) was screened to obtain a full-length clone. The sequence of the longest of the three isolated cDNA clones (2361 nt) has been deposited in Genbank (Accession number AF498294). This cDNA was named *Irm*, Imprinted RNA near Meg3/Gtl2. Initial blast searches with *Irm* cDNA of the nonredundant Genbank database yielded only a single cDNA match to AK017440, an uncharacterized RNA that differs from *Irm* by alternate 5′ splice site choice in exon 8. Subsequent searches yielded partial matches to two additional RNAs, the noncoding RNA *Rian* [29] and the long form of MBII-343 snoRNA [30]. In addition, four snoRNAs are present in introns of *Irm* [54]. Conceptual translation of *Irm* revealed that it is a noncoding RNA as it only has several short ORFs that lack significant homology to any known protein as assayed by blast searches (data not shown).

Corresponding regions in *Irm* cDNA from 129S1 and CzechII/Ei mice were analyzed, leading to the identification of a single nucleotide polymorphism at position 1094A→T creating a NlaIII restriction site in the CzechII/Ei cDNA (Accession numbers: AF498295 and AF498296). RT-PCR was performed on RNA derived from individual PMEF lines with the following genotypes, 129S1, CzechII/Ei, 129S1×CzechII/Ei, or CzechII/Eix129S1. RT-PCR products were purified and digested with NlaIII restriction enzyme. The resulting restriction digestion pattern shown in Figure 7A demonstrates that this cDNA is indeed maternally expressed.

Since *Meg8* and *Irm* cDNAs overlap substantially and are both maternally expressed, one possibility is that *Irm* represents a 5′ truncated transcript derived from the *Meg8* promoter. An alternate hypothesis is that *Meg8* and *Irm* have distinct promoters. Even though our Northern results support the latter hypothesis, RLM-RACE was performed to identify the transcriptional start site of *Irm* [55]. In this procedure, RNA is incubated with calf intestinal phosphatase (CIP), leading to the 5′ dephosphorylation of tRNA, rRNA, and fragmented transcripts. After CIP inactivation, samples are treated with tobacco acid pyrophosphatase that catalyzes the removal the ^7^mG cap with the liberation of a free 5′ monophosphate. An RNA adapter is subsequently ligated and RT-PCR performed with adapter- and gene-specific primers. Sequencing of the resulting RT-PCR products revealed that the transcriptional start site of *Irm* is located twelve nucleotides upstream of the original *Irm* cDNA sequence (5′gccaatgatgac with *Irm* 1-120, data not shown). This site is identical to the *Irm* promoter and corresponding transcriptional start site predicted by a neural network based algorithm (http://www.fruitfly.org/seq-tools/promoter.html). Moreover, the 620 bp length of the first *Irm* exon supports that the fact that it is the first transcribed exon, as less than 1% of internal exons are longer than 400 bp [56].

Over 60 mouse ESTs corresponding to *Irm* RNA are present in the Genbank database, providing considerable insight into the *Irm* expression pattern. In adult mice, *Irm* ESTs are found in the pancreas, eye, testis, and different areas of the brain, including the hippocampus, hypothalamus, amygdala, and striatum. In mouse embryos, *Irm* ESTs are found in the developing pancreas, brain, heart, testis, optic vesicle, and the neural retina. Given the profile of *Irm* ESTs, *Irm* expression was evaluated using Northern and whole mount *in situ* hybridization techniques using a probe common to exon 1 of both *Irm* and *Rian* RNAs. Northern analysis on adult tissues (Figure 7B) revealed high levels and multiple transcripts for *Irm* RNA in the brain. Significantly lower *Irm* expression was detected after longer exposure times in adult mouse testis, stomach, skin, heart, and muscle. Northern analysis of embryos at different developmental stages revealed that *Irm* expression is temporally regulated during development (Figure 7B).

As mentioned previously, *Irm* RNA partially overlaps a cloned noncoding RNA termed *Rian* for RNA Imprinted and Accumulated in the Nucleus [29]; however, *Rian* Northerns have not been reported to verify its existence and size. *Rian* is characterized by several peculiar features. Most notably, it contains three retained introns, lacks major polyadenylation signals at its 3′ end, and terminates in a region of genomic DNA that has 15 adenosine residues. Therefore, the identified *Rian* cDNA most likely represents an internally primed *Irm* RNA that is reversed transcribed from the polyA stretch and does not exist in the cell as a mature species. Since our probe for the *Irm* Northern would detect *Rian* equally well, our results demonstrate that *Irm* is the major transcript derived from the *Irm* promoter (∼2.5 kb) and raises doubts about the existence of *Rian*.

**Figure 8:**
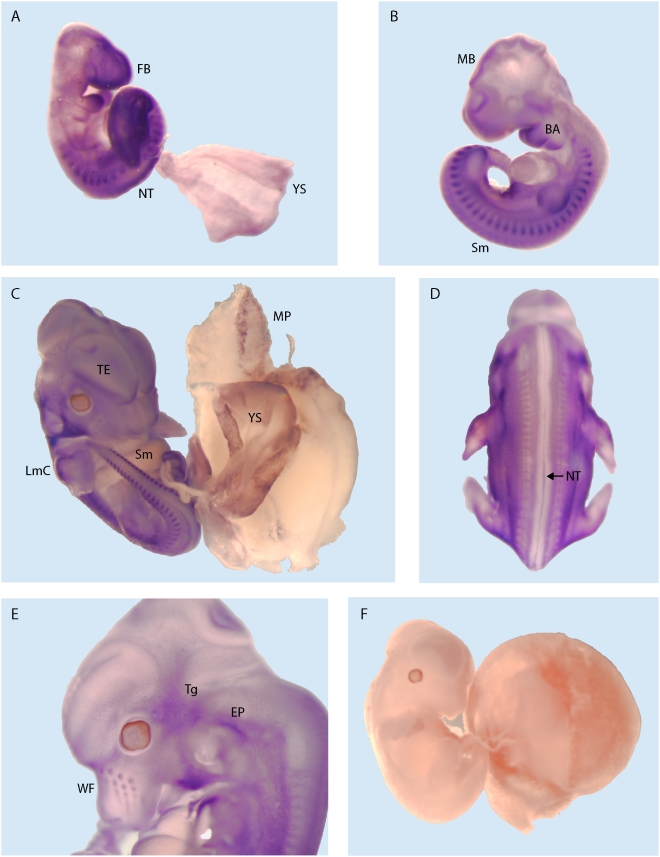
Tissue distribution of the *Irm* transcripts in the mouse embryo revealed by *in situ* hybridization. (A). On embryonic day 9.5 (E9.5), *Irm* expression is detected in the forebrain area (FB), first and second branchial arches (BA), in developing somites (Sm), and in the caudal neural tube (NT). A dispersed staining is observed in the extraembryonic tissues, particularly in the yolk sac (YS). (B). By E10.5 the brain expression domain shifts more in the midbrain area (MB) where the neuroepithelial lining shows the *Irm* staining. Similar to E9.5, expression persists both in the branchial arches and in the somites. (C). In E12 mouse conceptus, *Irm* transcripts could be also visualized in the forming limb cartilage (LmC), still clearly detectable in the telencephalon (TE) and caudal somites. The yolk sac (YS) and the maternal side of placenta (MP) are positive for *Irm* by *in situ* staining as well. The expression along the midline in the caudal neural tube (NT) is most likely associated with the floor plate area (D). In the head, prominent *Irm* transcript accumulation is observed in the trigeminal area (Tg), ear pinna (EP), and the developing whiskers hair follicle (WF, see panel E). (F). Hybridization with the sense *Irm* probe under the same conditions resulted in no specific signal.

The spatio-temporal distribution of *Irm* transcripts within the embryo was determined by whole mount *in situ* hybridization at different stages of development. Prominent and persistent expression of *Irm* RNA was detected in the somites from embryonic day 9.5 (E9.5) onwards (Figures 8A–C). Other tissues expressing *Irm* include the first and second branchial arches and the limb cartilage (Figures 8A–C). In extraembryonic tissues, *Irm* expression exhibited a weak, but scattered expression in the yolk sac and to the maternal side of placenta (Figures 8A and 8C). *Irm* transcripts are also found in the brain, most prominently in the forebrain (Figure 8A) and later at E10.5 in the layers enriched with neural precursors in the midbrain (Figure 8B). Other regions within the nervous system that were positive for *Irm* expression were the cranial ganglia (predominantly the trigeminal ganglion), telencephalon, and midline of the caudal neural tube (Figures 8D and 8E). In summary, Northern, whole mount embryonic *in situ* hybridization, and EST analyses demonstrate that *Irm* RNAs are predominantly expressed in the brain but is not strictly brain-specific. RT-PCR experiments on seven adult mouse tissues for the long form of MBII-343 yielded a similar conclusion [30] and is noteworthy since this RNA overlaps *Irm* exon 6.

### Imprinting of AK050713 and AK053394

**Figure 9:**
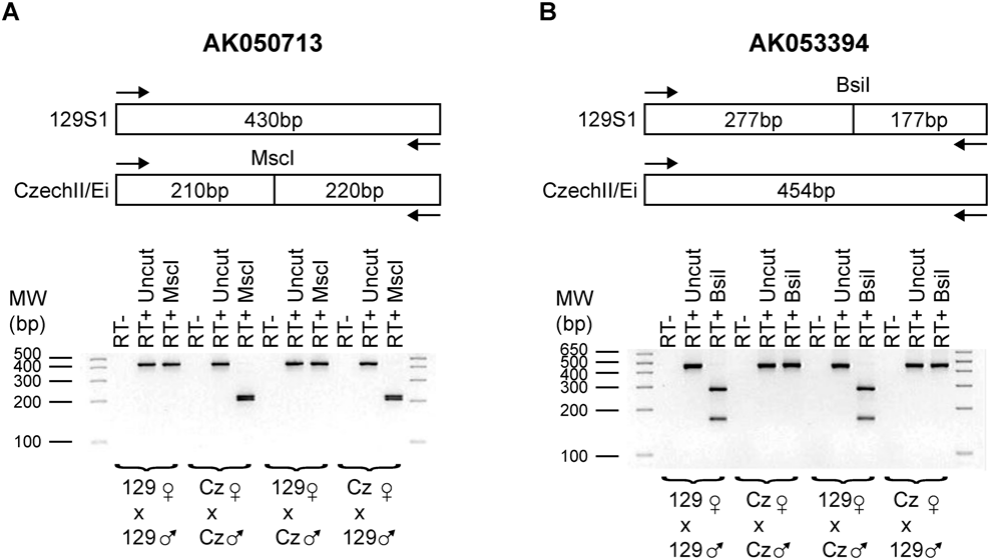
AK050713 and AK053394 are maternally expressed. (A) The AK050713 alleles contain an informative restriction site polymorphism in that a MscI site is present in the CzechII/Ei allele in comparison to 129S1. RT-PCR products with primers 784Up/1213Dn from the four intraspecific crosses were digested with and without MscI, verifying the imprinted expression of this gene. (B) Similarly, a BslI informative restriction site was utilized to demonstrate that AK053394 is maternally expressed using PCR primers 1647Up/2100Dn.

Multiple cDNA clones derived from Riken libraries map to the region between *Irm* and *Meg9*. Their accession numbers are from centromere to telomere as follows: AK050713, AK141557, AK163826, AK053394 and AK046809. For AK141557 and AK163826, RT-PCR with four sets of primer pairs did not detect a transcript in PMEFs or four adult tissues: brain, heart, muscle, and white adipose. These two putative cDNAs are unspliced, lack major polyadenylation signals, and end in a stretch of polyA mouse genomic sequence, suggesting that they may represent genomic DNA contaminants. For AK050713, we found that this transcript is maternally expressed in PMEFs (Figure 9A). Likewise, AK053394 is expressed exclusively from the maternal chromosome in PMEFs (Figure 9B). AK053394 is a spliced transcript with two exons and contains the entire sequence of AK046809.

### *Meg9/Mirg*: Imprinting, Expression, cDNA Cloning and MicroRNAs

**Figure 10:**
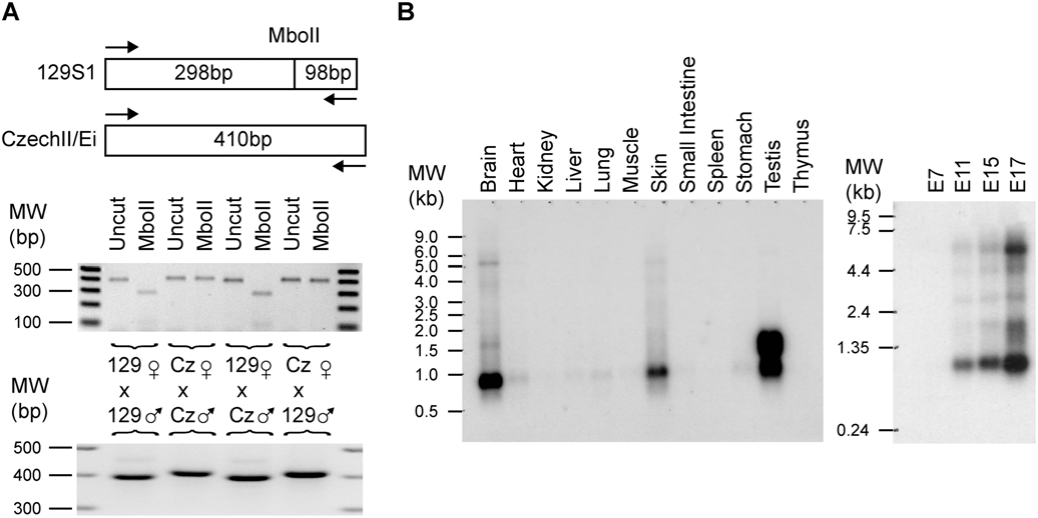
*Meg9* transcripts are derived from the maternal allele and are widely expressed in adult tissues. (A) *Meg9* alleles between 129S1 and CzechII/Ei differ by a polymorphism that abolishes a MboII restriction site in the CzechII/Ei allele. Moreover, the CzechII/Ei allele harbors a fourteen nucleotide long insertion. RT-PCR (bottom) and MboII restriction digestion (top) of the *Meg9* RT-PCR products demonstrated that this gene is maternally expressed. (B) Northern demonstrate that *Meg9*, like *Dlk1* (see Figure 3B), is widely expressed in adult tissues and that the abundance of its transcripts increase as a function of embryonic age.

Several spliced mRNAs and ESTs such as AK013406, CB193969, and BQ175773 are located approximately 73–88 kb downstream of the 3′ end of *Irm*. Sequences corresponding to AK013406 were determined for 129S1 and CzechII/Ei mice (Accession numbers: AF498297 and AF498298). The two alleles differ by a 14 bp insertion that abolishes an MboII restriction site in CzechII/Ei mice. Utilization of this informative restriction site on RT-PCR products revealed that this gene is maternally expressed. We thus named it *Meg9* for Maternally expressed gene 9 (Figure 10A). A day 19 mouse embryo cDNA library was screened, resulting in the identification of three transcripts that are all longer than 3 kb (Accession numbers: EU434920, EU616812, and EU616813). These transcripts overlap the recently identified *Mirg* (MicroRNA containing gene) [34]. In comparison to *Mirg*, the longest *Meg9* clone has nine additional upstream exons and contains four more microRNAs: miR-382, miR-134, miR-668, and miR-485. The location of the 5′ end of *Mirg* indicated that this truncated transcript results as result of processing of the exonic miR-485 precursor. As Figure 10 shows, Northerns using a probe that would detect both *Meg9* and *Mirg* demonstrate that *Meg9/Mirg* is widely expressed in many adult mouse tissues with the highest levels being in the brain, skin, and testis. *Meg9/Mirg* expression is also regulated during embryogenesis.

In total, *Meg9* has thirteen microRNAs as shown in Figure 2 and Table 2. Since several hundred genes are now known that contain at least one microRNA (see http://microrna.sanger.ac.uk/sequences/), we propose that this transcript for the sake of clarity be referred to as *Meg9/Mirg* to avoid unnecessary confusion with the vast number of other microRNA containing genes. In this imprinted region alone, *Meg3/Gtl2*, *Anti-Peg11*, and *Meg8* also contain microRNAs as already presented. The name *Meg9/Mirg* has already been used in four previous publications [57], [58], [59], [60]. For the thirteen microRNAs that are contained in the *Meg9/Mirg* transcription unit, examples of genes that are predicted to be targeted by individual microRNAs as determined by miRanda are shown in Table 2. Some noteworthy mRNAs are *Atp2a2*, *Bmp1*, *Calm2*, *Camk2g*, *Ctcf*, *Dicer1*, *Dlk1*, *Fgf10*, *H19*, *Kras*, *Mycn*, *Myf5*, *Myog*, *Myot*, *Mypn*, *Obscn*, *Peg10*, *Plagl1*, *Pdlim5*, *Sgce*, *Trp53*, and *Vim*. Moreover, miR-134 was recently shown to regulate negatively *Limk1* [61], *Nanog*, and *LRH1* [62].

### Imprinting of *Dio3* and *Anti-Dio3*

**Figure 11:**
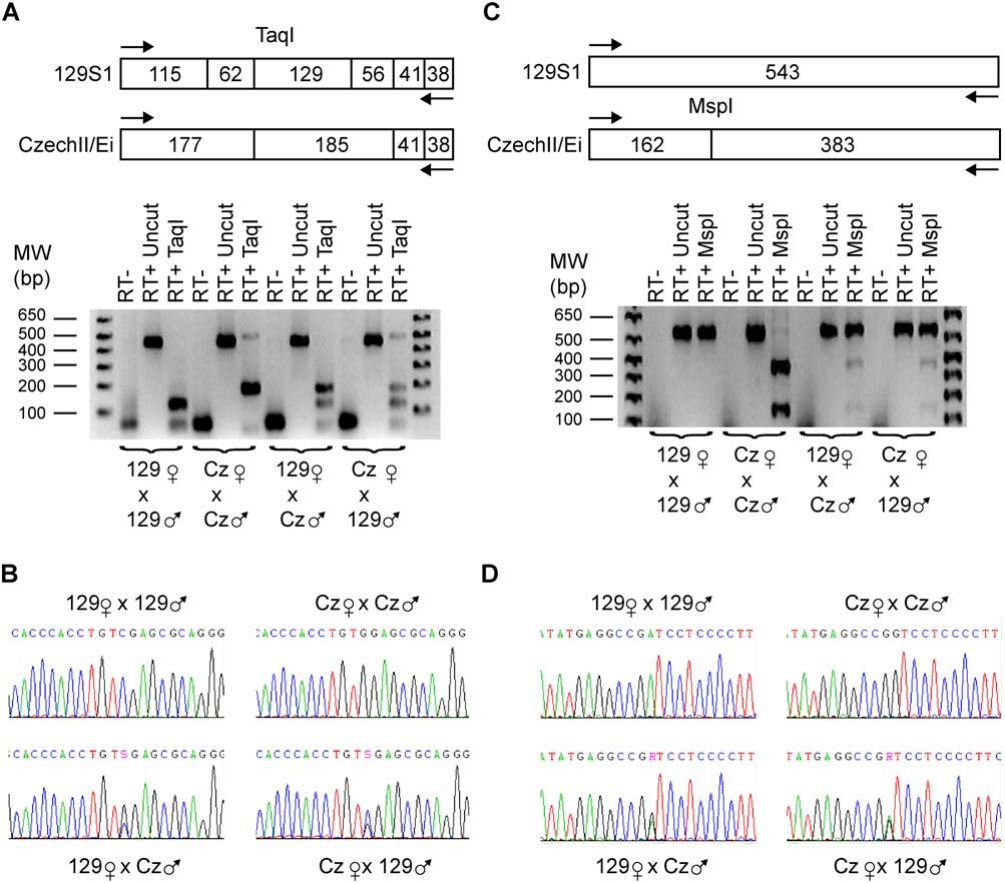
Allelic Expression of *Dio3* and *Anti-Dio3* (A) *Dio3* is an atypical imprinted gene in that the expression occurs preferentially from the paternal allele. Expression of the maternal allele occurs at roughly one-quarter the level of its paternal counterpart as evidenced by TaqI digests and (B) direct sequencing of RT-PCR products using *Dio3* 1216Up/1657Dn primers. (C) *Anti-Dio3* is biallelically expressed as evidenced by MspI restriction digests and (D) direct sequencing of RT-PCR products using *Dio3-AS* 654Up/1197Dn primers.

Previously, *Dio3* has been shown to be preferentially expressed from the paternal allele with the maternal allele expressed at roughly one-fourth the level of the paternal allele [31], [32], [33]. Our results confirm this finding (Figure 11A). In addition, the imprinting status of *Dio3-as* was investigated. *Dio3-as* was biallelically expressed without a preference for alleles (Figure 11B). This result is striking in that *Dio3* and *Dio3-as* appear to use the same promoter but are bidirectionally expressed.

## Discussion

Imprinted genes are characterized by allele specific expression and to a much lesser extent differential pattern of methylation. Several methods have exploited these characteristics to identify novel imprinted genes. Imprinted genes such as *U2af-rs1* [63], [64], *Grf1* [17], *Gnas* [65], and *Nesp* [66] were identified by differential methylation analyses. Most screens, though, exploit the differential expression of imprinted genes per se. Subtractive cDNA hybridization [18] and fluorescent differential display [29], [30] are also effective at identifying imprinted genes. In general, these techniques are quite labor intensive and require sufficient quantities of androgenetic and parthenogenetic embryonic material for analysis that is somewhat difficult to obtain. We utilized DNA microarray analysis on androgenetic and parthenogenetic PMEFs as an alternative approach to overcome these limitations. Six imprinted genes were among the forty most differentially expressed transcripts as determined by microarray analysis, revealing the relative efficiency of such an approach in identifying novel imprinted genes.

One maternally expressed transcript designated *Irm* (Imprinted RNA near *Meg3/Gtl2*) was a novel RNA that was cloned in its entirety. The *Irm* transcriptional start site as determined by RLM-RACE lies approximately 75 kb downstream from the end of *Meg3/Gtl2* on distal mouse chromosome 12. *Irm* noncoding RNA partially overlaps *Rian*, whose *in vivo* existence is questionable given its cDNA sequence and our Northern results. *Rian* cDNA contains at least three retained introns, lacks major polyadenylation signals at its 3′ end, and terminates at a stretch of 15 adenosines in the genomic sequence. Since *Irm* maps to a region with significant parent of origin effects in three species, an exhaustive screen for imprinted genes was undertaken for a 1.96 Mb region centered on *Irm*. We demonstrated that ten genes are imprinted. For six genes (*Dlk1*, *Peg11*, *Anti-Peg11*, *Meg8*, *Irm*, and *Meg9/Mirg*), we isolated and sequenced mouse cDNA clones. As Table 3 illustrates, the majority of these genes have not been identified as a cloned cDNA in any mammalian species. For mouse *Dlk1* and *Meg9/Mirg*, cDNA clones were described previously; however, our results increase our understanding of these genes. For *Dlk1*, we show by Northern, RT-PCR, and cDNA cloning that this gene has an abundant, alternatively polyadenylated transcript in the brain. Cross species comparisons reveal that this alternative polyadenylation site choice is a common characteristic of mammalian *Dlk1*. This finding clarifies the relationship between *Dlk1* and “DAT” by demonstrating that mouse “DAT” is not an independent gene but an alternate *Dlk1* transcript. For *Meg9/Mirg*, our cDNA contains nine upstream exons in relation to the original *Mirg* transcript. *Mirg* apparently is a truncated RNA generated when the exonic miR-485 precursor is removed from the *Meg9* transcript. Of note, it has been previously suggested that all the maternally expressed transcripts might generated by alternative splicing from the same gene [67]; however, our results exclude this possibility.

**Table 3:** Mouse cDNAs Cloned in This Study.

Mouse cDNAs cloned in this study	Previous reports	cDNA clones previously reported	Notes
Dlk1 Alternative polyA in brain	“DAT”, Dlk1-associated transcript (sheep and human)	None	“DAT” likely in sheep and humans to be alternatively polyadenylated form of DLK1
Peg11	Retrotransposon-like 1, Rtl1 (Mouse)	None (Computationally predicted Rtl1 ORF NM_184109)	Peg11 used to maintain consistency with mammalian orthologs as required by mouse gene nomenclature guidelines
	Peg11, paternally expressed gene 11 (sheep and human)	None	
Anti-Peg11	Rtl1-as (mouse)	None	Anti-Peg11 used to maintain consistency with mammalian orthologs as required by mouse gene nomenclature guidelines
	Anti-Peg11 (Sheep and human)	None	
Meg8	Meg8 (Sheep, human, and mouse)	None	Meg8 and not Irm contains miR-341 and the evolutionarily conserved miR-370
Irm	Rian (Mouse)	AB063319	Irm is the major product from Irm/Rian transcription unit. Rian RNA accounts for less than 5% of Irm RNAs as demonstrated by Northern. The cloned Rian cDNA (AB063319) contains three retained introns, lacks major polyA signals in its 3′ end, and its cDNA ends in a genomic stretch of 15 adenosines. Hence, the cloned Rian cDNA most likely represents a partially processed Irm RNA where reverse transcription was primed from the polyA stretch.
	long-form snoRNA-343 (Mouse)	AB076245	Irm Exon 6 overlaps the long form of snoRNA-343, suggesting that Irm gene encodes this snoRNA as well as MBII-426 and three others [54].
Meg9/Mirg	Mirg (Mouse)	AJ517767	Meg9/Mirg is used for clarity sake, since several hundred microRNA containing genes are known (see http://microrna.sanger.ac.uk/sequences/). This name has already been used in four previous publications [57], [58], [59], [60].
	Mirg (Sheep)	None	

The most significant implication of our results relates to how the identified imprinted genes may contribute to the parent of origin specific phenotypes that are observed in three mammalian species. Mice with either paternal or maternal uniparental disomy (UPDs) for this chromosomal segment exhibit non-viability, skeletal abnormalities, and embryonic growth defects [68]. Specifically, paternal UPD leads to placentomegaly, costal cartilage defects and late gestational lethality, while maternal UPD causes retarded growth and perinatal death. A second imprinting effect was revealed by a gene trap-LacZ insertion upstream of *Meg3/Gtl2* that causes dwarfism in mice inheriting the *Gtl2^LacZ^* mutant allele from their father [24], [69]. These mouse mutants phenocopy many features associated with human individuals that harbor UPD for the syntenic region of chromosome 14. Patients with UPD14 have numerous pathologies including cardiomyopathy, dwarfism, short limbs, mental retardation, scoliosis, musculoskeletal abnormalities, and intrauterine growth defects [70], [71], [72]. In addition, human 14q32 was recently identified as an imprinted modifier for bipolar affected disorder [73], delineating another potential role for this chromosomal segment in disease. In sheep, this region has been implicated as the locus responsible for the *callipyge* (“beautiful buttocks”) mutation that causes skeletal muscle hypertrophy when a single mutant allele of paternal origin is present [74]. This collection of phenotypes clearly illustrates the importance of developing a better understanding of this imprinted cluster.

Each of the three paternally expressed, protein coding genes in this imprinted region has been knocked out in mice. As previously discussed, *Dio3* is an atypical imprinted gene in that the paternal allele is preferentially but not exclusively expressed. A homozygous knockout of *Dio3* has a pronounced phenotype characterized by partial lethality (∼25%) and a 65% reduction in size at weaning age that persists throughout life [75]. Heterozygous mice where the mutated *Dio3* allele is inherited from the father are viable and are subtly growth retarded [32]. *Dlk1* knockout mice are characterized by 50% lethality by two days of age [76]. These mice have both prenatal and postnatal growth retardation with survivors exhibiting accelerated adiposity. Finally, mouse deletions of *Peg11* and several of its associated antisense microRNAs have recently been described [77]. Upon either maternal or paternal inheritance of the knockout, mice are characterized by a lethal phenotype that is mouse strain-specific. Upon paternal inheritance of the knockout, *Peg11* expression is lost while the maternally expressed microRNAs were unaffected in their expression. Maternal inheritance of the knockout results in loss of microRNAs that are antisense to *Peg11*, leading to elevated *Peg11* expression. This finding suggested that loss or overexpression of *Peg11* protein is important; however, an alternate hypothesis that can not be excluded is that *Peg11* mRNA is important instead as it serves as a functional sink to limit the action of several microRNAs. In summary, one can envision that the observed lethality for mouse mUPD12 is caused by loss of at least two if not all three protein coding genes in this region.

For mice with pUPD12, the identification of the responsible for the pathology is far more elusive. Maternal inheritance of the IG-DMR knockout upstream of *Meg3/Gtl2* leads to an absence of all investigated maternally expressed noncoding RNAs and is a fully penetrant perinatal lethal [78]. The significance of noncoding RNAs in imprinting, as well as other cellular processes such as transvection and RNA interference is well documented [79], [80], [81]. Many possible roles could be envisaged for *Meg3/Gtl2*, *anti-Peg11*, *Meg8*, *Irm*, and *Meg9/Mirg* genes. The significance of some of these genes as microRNA hosts will be discussed extensively later. Another role that these genes may have is in transcriptional regulation as these genes may compete for shared regulatory elements such as enhancers. The developmental expression pattern of *Irm* is strikingly similar to that found for *Meg3/Gtl2* [24], suggesting that these genes may be coordinately regulated during embryogenesis to at least day E12.5. We showed by Northern analysis that *Dlk1*, *Meg3/Gtl2*, *Meg8*, *Irm*, and *Meg9/Mirg* are all highly expressed in the adult mouse brain, although they differ somewhat in their relative expression levels in other tissues. These results suggest that these genes may be coordinately regulated. Validating this hypothesis, Su and colleagues performed large scale transcription profiling using microarrays on 61 mouse tissues, showing that *Dlk1*, *Meg3/Gtl2*, *Anti-Peg11*, *Irm*, and *Meg9/Mirg* are in a genomic region of coordinate gene expression [82]. Further experimental evidence supports this conclusion as the sheep *callipyge* (“beautiful buttocks”) mutation leads to altered expression of the five genes investigated in this imprinted cluster [23], [83].

The expression pattern of *Irm* and its coordinately regulated noncoding RNAs in this region is consistent with these genes contributing to the mutant phenotypes mentioned above. *Irm* expression, as determined by EST analysis, occurs in both the developing and adult retina, while uniparental disomy of syntenic human chromosome 14 in some individuals is associated with complete congenital achromatopsia [84]. Whole mount *in situ* data revealed that *Irm* RNA is present in the developing brain, branchial arches, somites, and limb cartilage. In adults, Northern analysis revealed that *Irm* levels are high in the adult brain and present, albeit to a much lesser extent in the heart, skin, muscle, and testis. The expression in the first branchial arch is particularly intriguing, as maternal UPDs of distal mouse chromosome 12 are associated with failures in ossification of the maleus and incus inner ear bones, derived from this embryonic anlage [68]. The *Irm* expression profile occurs almost exclusively in tissues that are adversely affected by documented parent of origin effects of this chromosomal region in sheep, mice, and humans.

The most obvious candidate for an essential noncoding RNA gene responsible for the pUPD phenotypes are microRNAs. In total, fifty-two microRNAs maps to this region and more than 80% have been identified in the orthologous human region. As previously reported, microRNA host genes and their associated microRNAs were shown to be in general coexpressed [85]. Given the coordinate nature of gene regulation for this region, the expression patterns of these microRNAs should be similar to each other and to their respective host genes. One of the most important observations about microRNAs is that each one apparently regulates on average ∼100 genes at the mRNA level [40], [41]. Therefore, it maybe somewhat naive to attribute a phenotype related to specific microRNA loss with overexpression of a single mRNA target. As Table 2 illustrates, the microRNAs in the *Meg3/Gtl2*-*Meg9/Mirg* region are predicted to target genes involved in placental, bone, and muscle biology. For example, six microRNAs are predicted to regulate *Dlk1* (underlined and written in bold in Table 2) suggesting another mechanism for interactions for allelic gene products besides *Peg11* and its antisense microRNAs. This observation is intriguing as it may account, in part, for the polar overdominance of the *callipyge* mutation. In addition, several well known genes involved in cancer initiation and/or progression are predicted to be microRNA targets. For example, loss of specific microRNAs may lead to overexpression of well known oncogenes such as *Bcl2*, *Fos*, *Igf2*, *Mycn*, and *Wnt1*. Likewise, microRNA overexpression could effectively downregulate tumor suppressors including *Brca1*, *Fhit*, *Rb1*, *Trp53*, and *Wwox*. This finding is particularly important given that a common Adeno-Associated Virus (AAV) insertion site in mice has recently been identified that maps between miR-341 and miR-370 in *Meg8* that causes hepatocellular carcinomas, suggesting that perturbed expression of microRNAs may be responsible. Ultimately, site directed mutagenesis in mice will help to resolve the relative importance of specific regions and genes within the *Dlk1-Dio3* region in the establishment of imprinting, in regulation of transcription, and in contributions to linked disorders. Of special interest, the role of microRNAs will need to be addressed in detail.

### 

#### Note Added to Proof

Recently, two additional partial mRNA sequences have been deposited in GenBank for the putative transcripts, *Mico1* (EF648170) and *Mico1-os* (EF648171) that map between mouse *Dlk1* and *Meg3/Gtl2* [86]. These cDNAs are 2012 bp in length and are exact reverse complements of each other.

## Supporting Information

Table S1 Sequences of Oligonucleotide Primers Used in this Study.
(0.10 MB DOC)

Table S2 Restriction site polymorphisms in cDNAs between 129S1 and CzechII/Ei
(0.07 MB DOC)
