# Supplementary material for: At Least Ten Genes Define the Imprinted Dlk1-Dio3 Cluster on Mouse Chromosome 12qF1
Source: PLoS One. 2009 Feb 5;4(2):e4352. doi: 10.1371/journal.pone.0004352 (PMC2632752; doi:10.1371/journal.pone.0004352)
Supplement: Table S1 — Sequences of Oligonucleotide Primers Used in this Study. (0.10 MB DOC) [file pone.0004352.s001.doc]

Table S1. Sequences of Oligonucleotide Primers Used in this Study.

| Gene | Primer sequences (5’–3’) |
| --- | --- |
| AA125385 (EST23) | 240 Up: agctcgggttgataagcaccttctc  491 Dn: ccatccctgctcacaatgacctg |
| AK014028 | 32 Up: gatgagactctgctcctccaaacgtc  598 Dn: ggctgtgtgcagctccttggtg  729 Up: ggaaatcataggctgccattcatgc  1205 Up*: gataatggtaactccagacatggcaa  1295 Dn: agagccaactatcattggaagcgatg  1612 Dn*: tgtagctgccctcaaaagccagtc |
| AK015759 | 65 Up: agctgagcctgcttccttgttgg  488 Dn: gaatgggactagggacacatggatagg  652 Up: cacaccaaattcccttctggtcacc  1174 Dn: tgtggctgtttcccacgagcac |
| AK019584 | 515 Up: tcatggatctgggaaagaggacctg  915 Dn: ccctggtggagtaggaccccaatac  1367 Up: tctttgcaccctgcttcatcaacc  1776 Dn: tgggatagctcttgggtaggagtgtg |
| AK044800 | 913 Up: ggtttctggggctttatgtaagctgg  1313 Dn: aaaggatgcggagggtagagcg  1636 Up: tgcaagtctggatcggttagaactgg  2041 Dn: cggcgagcttgactgatggacac |
| AK048151 | 1886 Up: tgacatcaccttagggtggaaggatg  2294 Dn: tctaacccctcatcagaaacatagctg  2227 Up: caagtccagaggcatctcacagagaa  2661 Dn: tgagcaaatgcacaggagtggtgtag |
| AK050713 | 784Up: tcctgacccagaatgatgacagtgc  1213Dn: tcagtattgcgacaacaaccaccactac |
| AK053394 | 1647 Up: tgttaggtgccttctctgcgtgc  2100 Dn: gctgacgcttccctgacaatcttgtag |
| AK147993 (BC007953-like) | 2656 Up: gcatcctcagtggtggcttcgac  3171 Up*: ggctgtgagtgttccccatgtgg  3175 Dn: ggtcaccacatggggaacactcac  3198 Dn: ggtcaccacatggggaacactcac  3225 Up: cgggttttgatgttcagtttccgc  3429 Up: agagctgctgggcttcagtgactc  3655 Dn: ttccaggaccaccagggctacac  3658 Dn: gagttccaggaccaccagggctac  3840 Up: tggtccttcggacagtgaggctc  3976 Dn*: gcaagttagaggaccagaggtgggtag |
| Akap12 (NM_031185) | 1369 Up: ggagcagaggtggaaggggatg  2037 Dn: cgtggaggacaaggtggcactc |
| Aldh1a7 (NM_011921) | 19 Up: cttgtgttccaggagccaaaccag  367 Dn: gattccattgtagccagcagcagac |
| Anti-Peg11 (NR_002848) /  Peg11 [EU434918] | Anti-Peg11 and Peg11  67Dn: gctgcttatgtccattagggcaaatc (67Dn) [3963 Up]  86 Up: gctctgaagcctctctggttccaac (86 Up) [3944 Dn]  365 Up: tagaaaggggtcttgaatagaaagc (365 Up) [3664 Dn]  477 Up: tgggttctgaggcttagggtgatagag (used 477 RT/North) (477 Up) [3552 Dn]  480 Up: gttctgaggcttagggtgatagaga (480 Up) [3549 Dn]  495 Dn: ccctaagcctcagaacccattctactt (495Dn) [3524 Up]  522 Up*: agccctcttcagggattccaagg (used 522 RT) (522 Dn) [3507 Dn]  572 Up: agggctctgaactcagcagttgtctc (572 Up) [3457 Dn]  661 Up: ttgaccttgtctatccctatcgccac (661 Up) [3368 Dn]  741 Dn: ggtgaaactgaacaagaaccttatg (741 Dn) [3288 Up]  776 Up#: cggtctggcgatggaactgag (776 Up) [3253 Dn]  812 Dn: tgttcactggacaaaactcagttccatc (812 Dn) [3217 Up]  822 Dn: caacatctactgttcactggacaaa (822 Dn) [3207 Up]  847 Dn*: aagtcctggtccgctttcggtatc (used 847 RT) (847 Dn) [3182 Up]  995 Dn: cccttcacaatgaactcatactctgac (995Dn) [3034 Up]  1052 Dn: gaagacacttggagagcatcgttcg (used 1052RT/North) (1052 Dn) [2977 Up]  1307 Dn#: cggcgtcttttacgagtctggg (1308 Dn) [2721 Up]  Peg11  26 Up#: gtgaccaagtctcgctctcg (NA) [26 Up]  54 Up: ctaacagtggttttggtctccac (NA) [54 Up]  259 Dn#: ctggtgtctcttcaacggtgtt (NA) [259 Dn]  503 Up: cccaccaggaaatgcaagagctg (NA) [503 Up]  1022 Up: gctttggttcaaacccagagtcgag (NA) [1022 Up]  1536 Up: tcgcagagactaccatgagtttgtcg (NA) [1536 Up]  1761 Dn: cagggtctgccgatactcaaacttttc (NA) [1761 Dn]  1988 Up: gagaagagcattactcaggctgccac (NA) [1988 Up]  2568 Up: cggattgccacacacatactcagacc (NA) [2568 Up]  4609 Up: gctgtcctggtgatgttgagagcaag (NA) [4609 Up]  4737 Dn: actactcgcaatgagggtcgag (NA) [4737 Dn]  4924 Dn: actcacacagggtcaaccagaac (NA) [4924 Dn]  5090 Up: cgacgtgcaagaggacctgtttg (NA) [5115 Up]  5531 Up: ctagccaactatcagcgtcccagaac (NA) [5531 Up] |
| Aquaporin 1 (NM_007472) | 944 Up: aggagtatgacctggatgctgacgac  1378 Dn: gcacaaagcgacattggcagaatc |
| AY077457 (Dio3-AS) | 378 Up: cccactttctgcctggactcttcttg  497Dn: ctgggaggtctgctgggcaaac |
| BC035527 (Weird AI132487) | 622 Up: tgagggtcactcctttgccacctac  674 Dn: ctcctcccgtacactggtcaccac  1266 Up: tgaggctgtgctgaggctcacg  1823 Dn: acaggtggtgtgggtatgcgatg |
| Begain (AK158915) | 243 Up: tgcccaggaggagctagacaagg  551 Up: cggacttccaacagcgcgtgag  674 Dn: ttctccagcactttggcgatgac  791 Up: tgcagcgacccagctctctactg  991 Up: cctccagctcctactccagcttcag  1109 Dn: agaactcgtcgcggctgctcag  1231 Dn: gtacggagacacagtccgagcaaag  1660 Up: agcaggctctcatgcctccaaac  2077 Dn: tccccatcactggctgcatagc  2259 Dn: tgtccttacggctcagcccagag  243 Up: tgcccaggaggagctagacaagg  674 Dn: ttctccagcactttggcgatgac  791 Up: tgcagcgacccagctctctactg  1231 Dn: gtacggagacacagtccgagcaaag  1629 Up*: tgagcctcagtcccagtcgttcg  2256 Dn*:ggctgccacccagaatctagcg |
| BG088109 (Dio3-AS) | 121 Up: agcactcacagggtccttctctg  233 Dn: gaaagttaggtagctggtctctcccg |
| BU055945 (part of spliced AK044800) | 11 Up: catctccccttcttgtgaggatgctac  450 Dn: cacagatccacctacctctgcctcc  291 Up: tgcgaagacctagaagatgaggcttg  730 Dn: cgacatgaatcacggacatcaagttagc |
| Casd1 (BC018542) | 1008 Up: caatcctaacaacaaactcttcgggc  1499 Dn: aacagtctctcattccacagcacgg |
| CB195177 | 19 Up: cagggggctggtgattcctgag  181 Dn: aatccgaggtgctgggtgtcatc  473 Up: caatgctgtgtacctgatggatggtt  699 Dn: tgccagccacgctctcattagaac |
| CD34 (NM_133654) | 1548 Up: ggctatgtagcacttgccctggacc  1719 Up: gcaagggtacagccagaaaagtgc  1945 Dn: tgaaccaatgagatttctgtgtgggc |
| Cdkn1a (NM_007669) | 964 Up: cctgcctggttccttgccacttc  1236 Dn: agagacccacaggagaggtgggctc |
| Col1a2 (nm_007743) | 2367 Up: ggtgctaaaggagagaagggaaccaaag  3023 Dn: aatgctgccagggtaaccacgc |
| Cyclin B1 (AK136259) | 10 Up: gcggaggaacggctgttagtgtttag  476 Dn: cgggcttggagagggattatcaacc |
| Depdc1a (NM_029523) | 2431 Up: acctacaatcaagcaaccgatgctg  2655 Dn: aggcacttagggcagatccagtagc |
| Dio3 (nm_172119) | 622 Up: gggtcaccacagattcaccctatgtc  836 Up: cggttaccaggtgtctgagttgcg  1190 Dn: tggtgtcagaaagtccagcaggtaaa  1216 Up: gaaagtccccagcatttcacagctc  1487 Dn: gcccagttagaggcggttcgtc  1657 Dn: acctcgcagattgattccccaag |
| Dio3-AS (AY283131) | 372 Up: tgtgaaagtggctcttctccctct  654 Up: gctggagagtctgagagacagatt  1197 Dn: caaacacagaaatgcatctgctgaac |
| Dkk3 (NM_015814) | 392 Up: aactatcacaatgagaccagcacggag  723 Up: gcaatgggaccatctgtgacaacc  1066 Dn: cacttcccctatgaagccaacatcttc  1395 Dn: tttctagcaacagcgagggacgac |
| Dlk1  EU434917  NM_010052 | 2 Up: ctggctttcttcccgctggac  34 Up: cttcgtggtccgcaaccagaag  193 Dn: ccataggtgctgtggccgaaag  274 Up: agggtcccctctgtgacaagtgtg  281 Up#: cctctgtgacaagtgtgtaactgc  317 Dn: gacacagccaggggcagtta  383 Dn: ttcgcagaatttcccgtcccag  388 Up: acgttcgggcttgcacctcaac  486 Up: ggaaaggactgccagcacaaggc  508 Dn: gccttgtgctggcagtcctttcc  631 Dn#: atctcacagaagttgcctgagaag  691 Up: acatcgggggtgacttccgttg  950 Up: cgtctttctcaacaagtgcgaaacc  968 Up: cgaaacctgggtgtccaacctgc  1027 F: tgttgcagtataacagcggcgagg  1101 Dn: tgaaagtggtcatgtcaatcttctcg  1237 Up: ggtggagtttgctctattgtgtggaatc  1314 Dn: gcatggcacacagcaacacaagac  1335 Dn: gggttcttagatagcgtggtagcatgg  1533 Up*: ggtcctcgtgaaaccataagagtgttg  1566 Dn: accactcactgtgaaaagggctaaactc  1638 Dn: ggtgtgagacttgatgtgagtgtgcg  1805 Dn: ggttggaggtgggggaatctcg  1887 Dn: ctgaatttgggagctagagaggactttg  1987 Up#: atcctggctgtacacttgatattga  2044 Dn: tccccattttgagaaacgctatcg  2114 Up: cctcttgagcaacccattccctacc  2238 Dn#: gaggtacactgaagtctgaaagcac  2613 Dn: cctaacggggacccaaggactg  2757 Up: ggtcatcagccattggtcatacgc  2829 Up: cccaggttcatttcacccaaagc  2884 Up: tcattcattaccttcagcccaagcc  3366 Dn: cacggctgctctccacaacagg  3428 Dn: tggatggaggaggagttgctaagagaac  3511 Dn: gctccaagaacactcactgccttacac  830 Up: acccaggtgagcttcgagtgtctg  956 Dn: tcaggcggtaggtgagcccatag  1274 Dn: tgaaagtggtcatgtcaatcttctcg |
| Dync1h1 (AY004877) | 1706 Up: cgccaacaccattgaggaggtg  2154 Dn: ccactcgcttcatgtaggcagtgag |
| Glyco49b (U05265) | 376 Up: cttcaccactggagcatacccacac  773 Dn: cgagatgataaagtttcgccctcagg  5540 Up: aggtctgatggggagtgtggtgg  5840 Dn: ggggcaatgtgtggttcttagtgg |
| Gpm6a (NM_153581) | 121 Up: tccagacccctttccactcccc  285 Dn: tgtttgcttagagactggctgtcaagtg |
| Grb10 (NM_010345) | 270 Up: atgaacaacgatattaactcgtccgtgg  770 Dn: cagcttctccatctgtgaagtgggc |
| H2afz (NM_016750) | 60 Up: tcttcctcgctcgtcggagcttc  556 Dn: ccactggaatcaccaacactggacag |
| Hat1 (NM_026115) | 510 Up: aaggctgacatgacatgtagaggctttc  758 Dn: agcatctgacttacacgtggccg |
| Igf2R (NM_010515) | 1850 Up: ttacactgatggtgatgactgtggcagtg  2194 Dn: tggcaggcccccgagtttgactgac  2818 Dn: atcctggtgctgtaggtggtgagctgg |
| Irm (AF498294) | 19 Up: gtcacggacgatgactacgtcactagag  46 Up: ggtctgaggtccatagcagaagatgc  120 Dn: tgtatgcacatgcctgacatgcaac  158 Dn: ctactatgaaatggtgagagtgcatgg  197 Dn: cctttccgtgcatggagatttgtatc  211 Dn: tcaatgccttaaatcctttccgtgc  338 Up: ggctgaagtcttcatgatggtcaagg  530 Up: gatgggtgctggcacatgatgtc  555 Dn: caagacatcatgtgccagcaccc  586 Dn: catggtgagagtgcaaccacggac  988 Up: tcggttgtgagattcctgctctgg  1058 Dn: ccacaaccaaggtgtacgcaacg  1140 Dn: aggagcaatggagaaagtgccaatc  1307 Up#: ttcacatcaatgggtggatcgtacc  1407 Dn: tcctcttccactgggttccctgag  1481 Dn#: tcagcagtcctcttgtgtctcgaag  2028 Dn: ggacttagtgaaaggctgaaggagct  2030 Dn: gaggacttagtgaaaggctgaaggagc  2241 Dn: cccaaacacacacccatttattcattc |
| Kitl (NM_013598) | 1046 Up: tcgcacagtggctggtaacagttc  1525 Dn: ctccatacagtggctgatgctacgg |
| Mad2l1 (NM_019499) | 17 Up: ccgtctgcggtgaggttggtag  441 Dn: tttcatcctgtatggctttctgggac |
| Mcm4 (NM_008565) | 261 Up: agcaggtcgtcacccaatcgg  644 Dn: cacatctgttccccaaatcacaagc |
| Mcm5 (NM_008566) | 2004 Up: ggctgatgtagaggaggcattgagac  2376 Dn: gtttggagcactgactgggagatagg |
| Meg3 (Y13832) | 267 Up: ggaacccactaccatacagaggaactcc  320 Up: agcgagggacaagcgacaaagag  578 Dn: ggacccttttgctcaggacattgttag  760 Dn: cccactaccccaaaagggacatctc |
| Meg8  AF498299  EU434919 | 1 Up: tgtgaatggagactagcctgctggtag  294 Dn: tgtgcttaggatcgtggggttgaag  376 Up: tgtgaatggagactagcctgctggtag  567 Up#: actcatcctgacgtgccaaaggtc  699 Up: aggggacagcaagtgtcagctcc  709 Dn: tgctgtcccctactgtacttctgctacg  906 Up: tctctgagatgaggactttgctatt  1051 Dn#: ggggatatggcagcatgaacctc  1214 Up: ttctattgtggacacgggacaccc  1238 Dn: tgggtgtcccgtgtccacaatagaaaac  1626 Up: gtctgcaggattcagatagtaatgg  1805 Dn: ctataagaacagagctgaccgtgac  2178 Up: ttcacatcaatgggtggatcgtacc  2352 Dn: tcagcagtcctcttgtgtctcgaag |
| Meg9 (EU434920) | 113 Up#: ttcatacaagagccctatcaggatt  268 Dn: gttgtttctggtccatcatttctt  359Dn#: cattaccaatctgcaagcactaaac  401 Dn: tgacaactggttgaccagaataaac  530 Up: cttctcttgattcatatgaggatgc  543Up: atatgaggatgctagtagccttggtt  742 Up: tgtgaactctagaaccaaggaagac  768 Dn: ctgtcttccttggttctagagttca  824 Up: gatttcctggacatgatggcaacttg  863 Up: gttcagtcaagctggtagagaagc  936 Dn: gacttacagctagctcaagcacact  1194 Dn: gtcttctttgagtggaattgatgct  1271 Up: ctgtggtcttaggggtgtatgttta  1445 Dn: gttggtatcatctcacagactctcc  1483 Up: ctaccacaggagcttccagaatac  1681 Dn: cctctctccaagtatcacacatga  1774 Up: actactgttcccaaggatgcctaat  1827 Dn: ccatgttcaccccagaagctgg  2162 Dn: gatcgaggaaagtgaagaagactg  2343 Dn: atggcgatgatggtggctaacg  2529 Up: aggagaggttgtctgtgatgagtt  2614 Up#: tcactcggcagtacataccaggtgtc 2704 Dn: cagggttccttgaacatccgctc  2788 Dn: gacttgttaatggccatagctctc  2883 Dn: ttgttgtcggttgtcgtaagcgag  3009 Dn#: ggtgggagttgaaacatgggtgag |
| Mpeg1 (NM_010821) | 428 Up: gggacgggtgatggacttgacatac  759 Up: gggtttacgaaggcacttatggacatc  938 Up: ccagaatagccagaacaccgtgacc  1115 Dn: ggtgatgcctggatagaagggaacc  1177 Dn: ggcaagccagcacggtctattg  1648 Dn: ccagcagccacacaccaataagc |
| Mtap (NM_024433) | 543 Up: aggacttcggtgccattcaaagg  774 Dn: aaccccatccactgacactgcttc |
| Onzin (AF263458) | 2 Up: cactcaacccagacccacaggacc  152 Up: ccagcctgtgtgattgcttcagtg  430 Dn: gccatccagctccttagaaagcg  516 Dn: tccatcccatctcagttgccaaag |
| Origene Vector Primers | OriVec 916Up: taagcagagctcgtttagtgaaccgtc  OriVec 1056Dn: cacatgttcaggaaacagctatgaccg |
| Peg3 (NM_008817) | 4430 Up: cgaagccaatgtcctcatcccac  4956 Dn: cccgttcaatgtagccagagcactc |
| Picalm (NM_146194) | 1618 Up: tagatgctgttgaggatgccattcc  1997 Dn: gccaagattgcccacaaggttg |
| Plac8 (NM_139198) | 315 Up: cggcattcctggatctatttgtgatc  696 Dn: agcaaaatcttttatttggtttctacc |
| Postn (NM_015784) | 1017 Up: agggtgcgaaggggacagtatctc  1513 Dn: tctctgctggttggatgatttctcg |
| Ppp2r5c (NM_012023) | 890 Up: tgaagtccctgagtgtctaccatccc  973 Up: gtggtaatggcacttctcaaatactggc  1651 Dn: cttccaaggctttctcggtgtgg |
| RacGap1 (NM_012025) | 1060 Up: aactgacacagacaatttgggcacac  1210 Up: tcgtttggtctcccatccagaatg  1841 Dn: gctccaccaccttgagctgacg  1944 Dn: ggtgtgcgtggtgttgagtttcc |
| RLM-RACE | 5’ RACE Outer: gctgatggcgatgaatgaacactg |
| Sgce (NM_011360) | 1146 Up: ccaaaaacagagagattgcatggcc  1260 Dn: aacggcatgttggtgctgtcgtag |
| Slc25a29 (BC006711) | 466 Up: cctggactgcctggtgcagatttac  1038 Dn: gaggagccataccctctgttgtcaag  1123 Up: cctcagacatggtacattcaggaagg  1634 Dn: ctggcagtaagcgagtcagacatcag |
| Slc9a3r1 (NM_012030) | Pre-Exon1 UpA: cgccccactctcctgattggttag  Pre-Exon1 UpB: acttccacttcctgggctctcttttc  90 Up: ggtgctcagctcccaactc  264 Dn: ggaagccgtagccatttggacc  390 Dn: tctccacattctcaccgttcacctc |
| Tcn2 (NM_015749) | 601 Up: cctcagcatcctggcactgtgtg  1151 Dn: cactggcgaccttcagcgtgac |
| Tenascin C (NM_011607) | 5419 Up: ccatccacccactactcagcaagg  5728 Dn: ggtctccaaacccagcagcatagg |
| Thy1 (NM_009382) | 1349 Up: gcagaagaagacaaggagccagaactc  1669 Dn: gtattatacatggtgatgggacagg  After pA Dn: gacagacgccaggacacagaagagac |
| Wars (NM_011710) | 338 Up: gatgccacaaaagccagcgagg  745 Dn: tgcctgctccagagtcaagtccttc |
| Yy1 (NM_009537) | 122 Up: tctacatcgccacggacggctc  155 Up: ccgagatcgtggagctgcatg  748 Dn: ccagggtcttgatctgcacctgc  779 Dn: gaggaccacatggtgaccgagaac  1190 Up: gcttttcactggacttcaatttgcg  1599 Dn: tgttgggaccacactttacaaaaacac  1755 Up: ttggggatatgcttagtaatgctgtg  2183 Dn: tgttgccctttctgttacacggatg  Pre-Exon2 37 Up: aggagttccaaaggattgaggacaca  Post-Exon2 498 Dn: gcacagctatcaccaaaaccgtttg  Pre-Exon4 87 Up: tctcagtttgcattgtgaaccactaa  Post-Exon4 421 Dn: agcagaccttcagagagctatggaa |
| Zac1/Plagl1 (NM_009538) | 1781 Up: atcctgttcctacctcatatgc  2244 Dn: ctggatctgcaactgaaactgtgg |
