# Supplementary material for: At Least Ten Genes Define the Imprinted Dlk1-Dio3 Cluster on Mouse Chromosome 12qF1
Source: PLoS One. 2009 Feb 5;4(2):e4352. doi: 10.1371/journal.pone.0004352 (PMC2632752; doi:10.1371/journal.pone.0004352)
Supplement: Table S2 — Restriction site polymorphisms in cDNAs between 129S1 and CzechII/Ei (0.07 MB DOC) [file pone.0004352.s002.doc]

| Supplemental Table S2. Restriction site polymorphisms in cDNAs between 129S1 and CzechII/Ei | | | | |
| --- | --- | --- | --- | --- |
| Gene Name | Accession  Number | Region Investigated | Polymorphism (129→Czech) | Restriction Enzyme  (129→Czech) |
| AK050713 | AK050713 | 784-1213 | 993A→G | No site→MscI |
| AK053394 | AK053394 | 1647-2100 | 1924C→T | BslI→No site |
| *Anti-peg11* | AK018276 | 477-1052 | 707C→T | No site→NlaIII |
| *Asb4* | AK076004 | 1330-2045 | 1521T→C | No site→SphI |
| *Cdkn1c* | AF160190 | 1076-2088 | 1684A→G | No site→TfiI |
| *Grb10* | BC016111 | 1561-2643 | 2441C→T  2467G→A | No site→PstI  No site→AccI |
| H19 Fetal Liver RNA | NM_023123 | 1261-1662 | 1304C→deleted  1631A→G | No site→BsmA1  No site→BglI |
| *Igf2r* Exon 14 | L22109 | 1-106 | 41T→C | BglII→No site |
| *Irm* | AK017440 | 941-1092 | 1046A→T | No site→NlaIII |
| *Meg3/Gtl2* | Y13832 | 267-578 | 364T→G | No site→Alw26I |
| *Meg8* | BF152417 | 119-295 | 234T→C  283G→A | DdeI→No site  HgaI→No site |
| *Meg9/Mirg* | AK013406 | 26-418 | 124A→T  395→+14bp Insertion | HpyCH4V →No site  No Site→MboII |
| Delta-like homolog 1 (*Dlk1*) | D16847 | 470-817 | 692T→C | DraIII→No site |
| *Dlk1* Alt. pA “DAT” | EU434917 | 1533-2044 | 1636G→A | EcoO109I→No site |
| *Dio3* | NM_172119 | 1216-1657 | 1332C→G  1523C→T | TaqI→No site  TaqI→No site |
| ε-Sarcoglycan (*Sgce*) | AF103877 | 140-1421 | 1232C→T | No site→NlaIII |
| *Igf2* | NM_010514 | 929-1726 | 1225C→T | DdeI→No site |
| Necdin (*Ndn*) | NM_010882 | 643-781 | 704C→A | SacII→No site |
| MyEF-3-like/*Peg10* | AF302691 | 1844-2197 | 1995T→G | MseI→No site |
| *Peg3* | AF038939 | 3860-4325 | 3982C→G | No site→FauI |
| *Zac1* | AF147785 | 1474-3064 | 2351C→T | No site→NsiI |
| *Slc25a29* | BC006711 | 466-1634 | 634T→C | No site→Bsp1286I |
| *Casd1* | BC018542 | 1008-1499 | 1435G→C | DdeI→No site |
| *Col1A2* | X58251 | 2304-4158 | 2788G→C  3517A→G  3838C→A | No site→MspI  BsmAI→No site  No site→BseGI |
| *Dync1h1* | AY004877 | 1706-2154 | 1790G→A | No site→StuI |
| *RacGap1* | AB030252 | 1094-1725 | 1418C→T | FspI→No site |
| *Mpeg1* | L20315 | 330-1551 | 787T→A  1108G→A | No site→AlwNI  AluI→No site |
| *Plac8* | AF263458 | 152-516 | 483T→C | No site→Tsp509I |
| *Postn* | NM_015784 | 1016-1517 | 1194A→T  1398A→C | Tsp509I→No site  No site→AciI |
| *Ppp2r5c* | AB055635 | 740-1384 | 927G→A | No site→Tsp509I |
| Tryptophanyl tRNA synthetase (*Wars*) | BC003450 | 337-745 | 370G→A | No site→TspRI |
| “Begain” Biallelic |  | 2017-2644 | 2282C→T | No site→BstXI |
| “BC007953like” Biallelic |  | 767-1993 | 1683T→C | No site→BsaAI |
| *Ppp1r9a* Biallelic |  | 5638-6330 | 3’ 388A→G  3’ 665C→T | No site→MspI  No site→AccI  RsaI→No site |
| “ASDio3” |  | 5958-6437 | 5984C→T | No site→BlpI |
| “Dio3AS” Biallelic |  | 4489-5190 | 4952A→G | No site→MspI |
| Anti-Dio3 | BG088109 | 121-232 | 198T→C | LweI→No site |
